# Research on the Tail Risk Spillover Effect of Cryptocurrencies and Energy Market Based on Complex Network

**DOI:** 10.3390/e27070704

**Published:** 2025-06-30

**Authors:** Xiao-Li Gong, Xue-Ting Wang

**Affiliations:** 1 School of Economics, Qingdao University, Qingdao 266061, China; m18953280729_1@163.com; 2 Laboratory of Complex Economic Systems and Digital Governance, Qingdao University, Qingdao 266061, China

**Keywords:** cryptocurrency, energy market, complex network, tail risk spillover, conditional quantile

## Abstract

As the relationship between cryptocurrency mining activities and electricity consumption becomes increasingly close, the risk spillover effect is steadily drawing a lot of attention to the energy and cryptocurrency markets. For the purpose of studying the risk contagion between the cryptocurrency and energy market, this paper constructs a risk contagion network between cryptocurrency and China’s energy market using complex network methods. The tail risk spillover effects under various time and frequency domains were captured by the spillover index, which was assessed by the leptokurtic quantile vector autoregression (QVAR) model. Considering the spatial heterogeneity of energy companies, the spatial Durbin model was used to explore the impact mechanism of risk spillovers. The research showed that the framework of this paper more accurately reflects the tail risk spillover effect between China’s energy market and cryptocurrency market under various shock scales, with the extreme state experiencing a much higher spillover effect than the normal state. Furthermore, this study found that the tail risk contagion between cryptocurrency and China’s energy market exhibits notable dynamic variation and cyclical features, and the long-term risk spillover effect is primarily responsible for the total spillover. At the same time, the study found that the company with the most significant spillover effect does not necessarily have the largest company size, and other factors, such as geographical location and business composition, need to be considered. Moreover, there are spatial spillover effects among listed energy companies, and the connectedness between cryptocurrency and the energy market network generates an obvious impact on risk spillover effects. The research conclusions have an important role in preventing cross-contagion of risks between cryptocurrency and the energy market.

## 1. Introduction

In recent years, the cryptocurrency market has undergone a dramatic evolution, attracting widespread attention from investors and researchers around the world [1,2]. Cryptocurrencies have played an increasingly important role in the global financial system [3]. As an emerging financial asset class, cryptocurrencies not only challenge the traditional financial system but also trigger in-depth discussions on their market behavior, risk characteristics, and investment returns [4,5,6]. Unlike traditional assets, cryptocurrencies are known for their high volatility and extreme returns, which gives them unique risk–return characteristics in investment portfolios [7,8]. Therefore, because of the complexity and uncertainty of the cryptocurrency market, it is important to conduct a systematic analysis of it for financial research.

Energy is an important strategic resource for countries, directly affecting their economic development and environmental sustainability. In responding to global challenges and promoting economic growth, the sustainable utilization of energy has become an important issue for global society. It is also crucial to control energy market risks. In China, one of the most populous countries in the world, energy consumption, financial development, and international trade have long-term dynamic connections [9], with a significant impact on the stability of the whole energy market. In previous research on the energy market, most studies have used energy stock indexes as research variables. However, this ignores the heterogeneity of different companies’ volatility in risk shocks [10]. As a result, this paper conducts a micro-level analysis of individual companies, examining the risk spillover effects between cryptocurrencies and listed energy companies.

Since energy prices are subject to international benchmark oil prices and associated policies, the stock values of energy companies tend to show strong connections. For example, the “shale revolution” in the United States changed the world’s energy landscape, causing international oil prices to plummet in the second half of 2014. The stock prices of Chinese listed energy companies also showed similar fluctuation trends. In recent years, with the support of government policies, China has achieved remarkable results in the development of new energy sources, such as wind power and photovoltaic power. Energy companies are also expanding their businesses in new energy fields to comply with development trends, so stock prices also show connections. In addition, due to the high degree of integration of the energy market, the industrial chains of different energy companies often overlap and have strong connections. Furthermore, energy companies show similarities when exposed to energy price fluctuations, geopolitical risks, and economic policy uncertainties. As a result, to prevent systemic risk spillovers, it is necessary to examine the tail risk spillover effect of the energy market from the standpoint of spatial network connection.

Nowadays, the connection between cryptocurrencies and energy commodities is becoming increasingly apparent [11], and energy market shocks have a great influence on the volatility of cryptocurrencies. Among them, cryptocurrency mining activities consume a lot of electricity due to high computing power. According to 2024 data, Bitcoin’s annual energy consumption exceeded 10 million megawatt-hours, which is equivalent to the total electricity consumption of some medium-sized countries. This high energy demand has raised concerns about environmental impacts, especially in regions where energy mainly comes from fossil fuels, such as China and the United States [12]. In addition, extreme event shocks have contributed significantly to the risk volatility of both the cryptocurrency market and the energy market. For example, Sino–US trade friction has had a great influence on cryptocurrency and the energy market [13,14,15]. Additionally, dramatic events like the COVID-19 shock and the Russia–Ukraine war have had varying degrees of impact on cryptocurrencies and China’s energy market due to their different scales [16,17,18,19]. Hence, it is crucial to examine the tail risk spillover effects that exist between cryptocurrency and the energy market.

As countries have gradually attached importance to the digital economy, China has followed the pace of the times and incorporated the development of the digital economy into its overall framework. Cryptocurrency, based on blockchain technology, has attracted widespread attention from scholars, policymakers, and financial regulators. In the early stage of cryptocurrency development, China dominated the field of cryptocurrency mining. Cryptocurrency mining activities consume a lot of electricity. Due to its lower electricity prices and the control of the global mining machine supply chain by the three major mining machine manufacturers, China once accounted for more than 65% of the global Bitcoin computing power. However, the prosperity of cryptocurrency also implies risks. Its decentralized characteristics make it difficult for the government and financial regulators to monitor and easily disrupt the normal order of the economy and finance. On 4 September 2017, the state issued the “Notice on Preventing the Risks of Token Issuance and Financing”, which explicitly prohibited cryptocurrency transactions in mainland China, killing 90% of the global Bitcoin trading speculation market. The price of cryptocurrency has been greatly impacted, and the decline in mining energy consumption has also had an impact on the related energy market in China. Based on the crackdown and withdrawal of domestic residual mines and the shift in energy policy, the 2021 policy clearly listed mining as a “high-energy-consuming and inefficient industry”. On 24 September, the “Notice on Further Preventing and Dealing with the Risks of Virtual Currency Trading Speculation” was issued in China. The crypto bans triggered a continuous plunge in Bitcoin’s global hashrate. It can be seen that China’s disposal of cryptocurrencies affects the global cryptocurrency market, and the withdrawal of cryptocurrencies will inevitably lead to the transformation of the coal-fired power industry. Furthermore, Chinese energy policy is tilted toward the new energy industry, and the pattern of China’s energy market will change as a result. In addition, since the academic community has proposed a distributed photovoltaic-driven mining model and foreign countries have begun to explore methods of mining using clean energy and waste energy, domestic seminars in 2024 began to discuss the compliance of cryptocurrency and new energy linkage. In short, the promulgation of the cryptocurrency ban cannot completely separate the cryptocurrency market from China’s energy market. On the one hand, the study of the risk spillover effect between the two can help the government and regulators to promptly discover problems and prevent risks. On the other hand, it can also identify new ways out for the compliance of cryptocurrencies and the full utilization of energy.

In the study of the cross-market contagion mechanism of risk, many scholars have discovered the significance of tail risk in the entire risk contagion [20,21]. When the market is hit, stocks with high tail beta suffer much greater losses than stocks with low tail beta [22], and risk prevention should be more focused on the thick-tail distribution [23]. When risks spread in the market through the connection network, the complexity of the network structure affects the internal associated nodes. Large-scale shocks may trigger systemic risks. Therefore, in order to more thoroughly measure the tail risk contagion effect, this paper employs the complex network method to examine the tail risk spillover effects of cryptocurrency and the energy market at various quantiles from a time–frequency perspective. It also examines the influence of network connection characteristics on the risk spillover index.

Given the intricate relationship between the cryptocurrency and energy markets, this study innovatively investigates the risk spillover effects between the energy market and a major cryptocurrency from a micro-level perspective of energy-listed companies. In the research process, it employs a GARCH model integrated with the Johnson-SU distribution to extract volatility from the logarithmic returns of cryptocurrency and energy-listed stocks. Utilizing complex network methodologies, the study combines the Quantile Vector Autoregression (QVAR) model with time–domain risk spillover networks and further innovates by integrating frequency–domain spillover networks to analyze evolutionary trends across different time horizons. Finally, recognizing the spatial interdependencies among energy-listed companies, the research further explores the influence of network topology indices and selected macro–micro indicators on risk spillover dynamics within energy markets.

The remainder of this paper is organized as follows. Section 2 introduces the literature review. Section 3 presents the theoretical model. Section 4 is a presentation and analysis of empirical results, which analyzes the tail risk spillover effect of cryptocurrency and China’s energy market from both static and dynamic viewpoints in the time–frequency domain under different market risk situations. Then, the spatial Durbin model is employed to analyze the impact of network structure characteristics on the spillover level of various time periods. Section 5 concludes this paper.

## 2. Literature Review

### 2.1. Research on Risk Contagion Between Cryptocurrency and Energy Market

With the frequent occurrence of severe events, the volatility of energy prices and the risk linkage of energy markets have attracted widespread attention. Research on energy markets has also concentrated on the study of national or regional energy indices [24,25], while there are few studies on the risk spillover effect of energy markets at the company level. Uddin et al. [26] studied the extreme uncertainty connectedness between 68 companies from four energy-related subsectors and concluded that the spillover effect in the renewable energy subsectors was the strongest. In a network of the top 20 global energy businesses identified by Platts global energy company rankings, Wu et al. [27] assessed risk connectedness using the VaR model. Compared with the use of comprehensive indexes for research, studying the risk volatility of the energy market from the micro level of energy companies can reduce the homogeneity effect brought by macro factors, so as to more clearly find the risk differences between micro individuals. Therefore, this paper selects listed energy companies as representatives of the energy market.

To date, a large number of studies have examined the risk linkage effect between cryptocurrencies and various financial assets [28,29]. Since activities such as cryptocurrency mining lead to electricity and other energy consumption [30], the connection between cryptocurrencies and energy has gradually been discovered [31]. Previous studies have put cryptocurrencies in the same framework as energy and other major commodities and found that cryptocurrencies have begun to impact the entire commodity market [32]. Ghabri et al. [16] found that there is a close connection between Bitcoin, stablecoins, and crude oil prices, and the impact of COVID-19 has affected the connection between the three. Okorie and Lin [33] found significant one-way volatility spillovers from the cryptocurrency market to the crude oil market. As for the research on cryptocurrency and listed energy companies, only Symitsi and Chalvatzis [34] demonstrated the volatility spillover relationship between Bitcoin and energy and technology companies. Hence, innovatively analyzing the risk spillover effect of cryptocurrency and energy markets from the perspective of representative cryptocurrency and listed energy companies can more comprehensively elucidate the risk contagion characteristics between cryptocurrency and energy markets.

### 2.2. Research on Tail Risk Spillover Model

Existing studies have found that the returns on financial assets often exhibit leptokurtosis, thick tails, and volatility clustering effects [35,36,37]. Typical GARCH can effectively characterize the volatility clustering and heteroskedasticity of financial time series [38], but it is difficult to capture the asymmetry of volatility. The EGARCH model proposed by Nelson [39] can better describe the long memory and leverage effect of volatility. The research by Ni et al. [40] also confirmed the significant advantages of the EGARCH model in characterizing the persistence and asymmetry of volatility. In addition, in the study of capturing the tail characteristics of financial assets, we found that the Johnson-SU distribution can characterize the peak and thick-tail properties of financial assets. The Johnson distribution system was obtained by Johnson [41] performing different transformations on stochastic variables that follow the standard normal distribution. Choi et al. [42] applied it to the estimation of univariate and multivariate GARCH models and discovered that the extreme tail shape of daily exchange rates and stock returns, as well as the entire shape of the conditional distribution, were better described by the SU-shaped distribution than by the normal distribution and Student-t distribution. As a result, this paper incorporates it into the volatility spillover model to reveal the tail risk contagion effect of cryptocurrency and China’s energy market.

In terms of characterizing the tail risk spillover of cryptocurrency and China’s energy market, Diebold and Yilmaz [43,44,45] introduced the spillover index model in the time domain based on the generalized forecast error variance decomposition (GFEVD) of the vector autoregression model (VAR). Next, this paper utilizes the rolling window method to capture the risk spillover time-varying estimation. Then, a directed weighted network was constructed, which has become a commonly used method for measuring spillover levels in recent years [46,47]. Simultaneously, researchers have shown that as financial shocks spread, they may produce different frequency responses. There is a time–frequency dependence between energy markets, and the DY method examines risk spillover effects solely from a time domain standpoint, which cannot represent the risk spillover volatility in different cycles [48,49]. Then, Baruník and Křehlík [50] divided the time domain risk spillover into several frequency bands and built the spillover index framework under the frequency domain. This framework is based on the spectrum representation approach of the GFEVD. It may be used to assess the risk spillover effects over various time periods and quantify the connection between diverse frequency response variables. Asadi et al. [51] examined the connection between the volatility of the coal, natural gas, crude oil, stock, and currency markets in China and the United States using the combination of DY and BK methodologies. They also provided suggestions for investors’ investment strategies. Furthermore, Mensi et al. [52] used a dual perspective of time and frequency domains to study the frequency dynamics of volatility spillovers between Brent crude oil and stock markets in the US, Europe, Asia, and five vulnerable EU countries. They found that the cost of long-term hedging is higher than that of short-term hedging.

In addition, both the DY and BK methods are estimated based on the conditional mean of volatility spillovers, but shocks that occur in various market situations do not spread equally. Research indicates that the conditional mean risk spillover index may somewhat undervalue the impact of unexpected event shocks under severe circumstances [53]. To investigate the risk spillover impact in various quantiles, Ando et al. [54] developed the quantile vector autoregression (QVAR) model, which is founded on the DY model. Chatziantoniou et al. [55] combined the QVAR model with the BK method and proposed a time–frequency analysis framework that can examine the volatility spillover impact of various conditional quantiles in different time periods [56,57,58,59]. As a result, this paper will construct an EGARCH–JSU–QVAR–BK framework to examine the risk contagion between the cryptocurrency and energy markets under extreme event shocks.

### 2.3. Complex Network Method and Network Topology Index Construction

Information theory was established by Shannon in 1948. Its core is to study the quantification, transmission, and processing of information. Information theory quantifies the statistical dependence between two systems through “mutual information” and has been widely used in sociology, political science, management, and other disciplines related to information dissemination in recent years. With the development of global Internet technology and communication technology in the 21st century, social networks and biological networks have shown “scale-free characteristics” and “small world effects”, and the theory of complex networks has emerged. After the financial crisis, the academic community gradually paid attention to the cross-market contagion of financial risks and began to use complex network methods to study information dissemination and risk contagion in financial markets [60,61]. At this time, the cross-application of information dissemination theory and complex network theory was developed [62]. The research focused on using structured analysis tools such as nodes, edges, and centrality to quantitatively study the diffusion mechanism and path of information in the financial system. The complex network method abstracts each financial variable into a network node, and the relationship is represented by the edge strength and arrow direction, which more intuitively shows the risk propagation path. In addition, the construction of the network topology index is of great significance to the study of risk contagion. The centrality of the node can reflect the systemic importance of the corresponding financial variable in the entire network, providing quantitative support for the early warning of financial system risks [63]. Consequently, this paper uses the complex network method to construct a risk contagion network diagram for intuitive analysis and calculates the network topology index for subsequent analysis of the factors affecting energy market risk spillover.

### 2.4. Research on the Spatial Spillover Effect of the Energy Market

Since spatial econometric models can measure not only the geographical distance between variables but also the economic distance between variables, more and more scholars have combined network analysis methods and spatial econometric models to study the risk diffusion channels of asset price volatility in the financial market from a broader perspective. Jiang and Jin [64] found that emotional shocks could be transmitted and amplified between companies through spatial effects based on Chinese stock market data from 2012 to 2018. Due to the mutual influence between energy companies in spatial geography and business dealings, more and more scholars have begun to pay attention to the spatial spillover effect of the energy market. Zhu et al. [65] studied the magnitude of extreme risk spillovers between stock returns of 124 energy companies around the world and used a spatial panel model that considered spatial heterogeneity between companies to explore the determinants of risk spillovers. As an indicator of the role and importance of an individual in the entire network, it is also of great significance to study its relationship with the tail risk spillover of the energy market [66]. Therefore, this paper innovatively uses the short-term, medium-term, and long-term energy market risk spillover matrix of the 0.50 conditional quantile (normal conditions) to measure the economic distance between listed energy companies and then uses the spatial Durbin model to explore the impact of network structure characteristics on risk spillover.

The main innovative contributions of this paper include the following aspects. (1) The non-normal ARMA–EGARCH–JSU model is used to characterize the leptokurtosis and fat-tail characteristics of the return distribution of stocks, and the volatility under extreme event shocks is extracted. (2) To derive the time–frequency risk spillover effect under various market situations, the QVAR model is integrated with the risk spillover network in the time and frequency domains, respectively. This study also examines the dynamic variation of the tail risk spillover between China’s energy market and cryptocurrency. (3) From the perspective of spatial connection, based on the network features in various frequency domains and the risk spillover matrix, the influencing variables of the energy market’s tail risk spillover are examined.

## 3. Theoretical Model

This paper innovatively constructs the research framework for tail risk spillover between cryptocurrency and China’s energy market. Primarily, considering the non-normal properties of financial returns under extreme event shocks, the stochastic volatility model with leptokurtic and thick-tailed characteristics is used to fit the return volatility of energy companies and Bitcoin stock prices. Furthermore, from the dual views of the time and frequency domains, the variations in tail risk spillover effects based on various quantiles are examined, and the risk cross-contagion features of various time periods under various risk states are analyzed. Ultimately, the influence of changes in the network connection characteristics of China’s energy market on the risk spillover effect is analyzed from a spatial perspective.

### 3.1. Leptokurtic Stochastic Volatility Model

Before applying the quantile time–frequency spillover framework to examine the tail risk contagion of cryptocurrency and China’s energy market, we must first obtain the volatility of Bitcoin and energy company stock prices that reflect changes in tail risks. The distribution of stock price returns often displays leptokurtic, thick-tailed, and biased distribution characteristics. Hence, the above atypical phenomena should be considered when extracting stochastic volatility. However, the GARCH model is not effective in characterizing the leptokurtic, thick-tailed characteristics of the variables. For this reason, this study establishes the ARMA–EGARCH–JSU model to obtain the stochastic volatility. The model’s particular form is as follows:

(1) $$\begin{array}{l} y_{t}=\varphi_{0}+\sum_{j=1}^{p}\varphi_{j}y_{t-j}+\sum_{i=1}^{q}\varepsilon_{t-i}+\varepsilon_{t}\\ \varepsilon_{t}=\sigma_{t}\upsilon_{t}\\ g\left(\upsilon_{t}\right)=\theta \upsilon_{t}+\gamma \left[\left|\upsilon_{t}\right|-E\left(\left|\upsilon_{t}\right|\right)\right]\\ \ln\sigma_{t}^{2}=\omega +\sum_{i=1}^{q}\alpha_{i}g\left(\upsilon_{t-i}\right)+\sum_{j=1}^{p}\beta_{j}\ln\sigma_{t-j}^{2} \end{array}$$

where *ω* > 0, E*_t_*_−1_(*g*(*υ_t_*)) = 0; the EGARCH model has no restrictions on the parameters *α_i_*, *β_j_* and introduces the parameter *γ* to explain the asymmetry of information shock, which broadens the application scenarios of the model. In order to capture the leptokurtosis and fat tail of return distribution, this paper assumes that the error term *ε_t_* of stock returns follows the Johnson-SU distribution, and its density formula is shown as follows.

(2) $$g(\varepsilon_{t}|\mu ,\sigma ;\gamma ,\delta )=J\cdot \phi \left(Z\right)=\frac{\delta }{\sigma_{t}^{2}\sqrt{1+{\left(\frac{\varepsilon_{t}-\mu }{\sigma_{t}^{2}}\right)}^{2}}}\phi \left(\gamma +\delta sinh^{-1}\left(\frac{\varepsilon_{t}-\mu }{\sigma_{t}^{2}}\right)\right)$$

where *Z*~*N*(0,1); *μ*, *σ*, *γ*, *δ* represent the location, scale, skewness, and kurtosis parameters, respectively; *ϕ*(·) is the probability density function of the standard normal distribution; and *J* represents the Jacobian determinant of the transformation, that is:

(3) $$J=\left|Z_{x}^{\prime }\right|=\frac{\delta }{\lambda \sqrt{1+{\left(\frac{\varepsilon_{t}-\mu }{\sigma_{t}^{2}}\right)}^{2}}}$$

### 3.2. Quantile Time–Frequency Spillover Network

This study illustrates the tail risk contagion from the standpoint of time and frequency domain after taking into account the tail risk components and computing the stock volatility of cryptocurrency and energy companies in China’s energy market. Then, the effect of varying magnitudes and cycles of volatility shocks on the tail risk contagion of cryptocurrency and China’s energy market is analyzed, and the dynamic estimation of total spillover and directional spillover is realized. Shocks of different scales can be captured at different quantiles of volatility. The larger the shock scale, the greater the volatility. Excessive market volatility leads to problems such as declining consumer confidence and reduced corporate investment and financing scale. Too low volatility leads to market laxity, and regulators will lack the ability to predict and prevent risks. Based on the above, this paper assumes that the 0.50 conditional quantile represents normal conditions, the 0.05 conditional quantile represents severe downward conditions, while the 0.95 conditional quantile represents severe upward conditions.

First, the specific setting of the *N*-dimensional QVAR model with a quantile level of *τ* in the time domain is as follows:

(4) $$x_{t}=\mu_{t}\left(\tau \right)+\Phi_{1}\left(\tau \right)x_{t-1}+\Phi_{2}\left(\tau \right)x_{t-2}+\cdots +\Phi_{p}\left(\tau \right)x_{t-p}+u_{t}\left(\tau \right)$$

where *x_t_* and *x_t_*_−*I*_ (*i* = 1,…, *p*) are the *N* × 1 dimensional endogenous variable vectors. The quantile level *τ* ranges from 0 to 1 inclusive, representing the change in the size of the shock. *μ_t_*(*τ*) is the *N* × 1 dimensional conditional mean vector, Φ*_j_*(*τ*) expresses the *N* × *N* dimensional coefficient matrix, and *u_t_*(*τ*) expresses the *N* × 1 dimensional error vector. For Formula (4), we use the Wold theorem to convert it into an infinite-order QVMA process:

(5) $$x_{t}=\mu \left(\tau \right)+\sum_{j=1}^{p}\Phi_{j}\left(\tau \right)x_{t-j}+u_{t}\left(\tau \right)=\mu \left(\tau \right)+\sum_{i=0}^{\infty }\Psi_{i}\left(\tau \right)u_{t-i}$$

Subsequently, this study uses GFEVD to show how each variable affects other variables and itself in the *H*-step forecast error variance at various quantile levels *τ*. Since the row sums of *θ_ij_*(*H*) are not equal to one, further standardization is performed to obtain Formula (7), at this time, $\sum_{i=1}^{N}{\tilde{\theta }}_{ij}\left(H\right)=1$, $\sum_{j=1}^{N}\sum_{i=1}^{N}{\tilde{\theta }}_{ij}\left(H\right)=N$.

(6) $$\theta_{ij}\left(H\right)=\frac{{\left(\sum \left(\tau \right)\right)}_{jj}^{-1}\sum_{h=0}^{H}{\left({\left(\Psi_{h}\left(\tau \right)\sum \left(\tau \right)\right)}_{ij}\right)}^{2}}{\sum_{h=0}^{H}{\left(\Psi_{h}\left(\tau \right)\sum \left(\tau \right){\Psi^{\prime }}_{h}\left(\tau \right)\right)}_{ii}}$$

(7) $${\tilde{\theta }}_{ij}\left(H\right)=\frac{\theta_{ij}\left(H\right)}{\sum_{k=1}^{N}\theta_{ij}\left(H\right)}$$

where ${\tilde{\theta }}_{ij}\left(H\right)$ quantifies the degree of spillover from variable *j* to variable *i* in the time domain across multiple quantiles, indicating the impact of the exceptional events from variable *j* to the forecast error variance of variable *i* for an *H*-step forward prediction at conditional quantiles.

Using Formula (6), this study determines the total spillover level and the directional spillover level under various conditional quantiles based on this foundation. The spillover-in index (*FROM*) measures the effect variable *i* derived from the other variables *j*, while the spillover index (*TO*) reflects the influence of variable *i* on other variables *j*. The net spillover effect (*NET*) of variable *i* is determined using Formulas (8) and (9). The influence of variable *i* on other variables *j* is larger (less) than the impact from other variables *j* if *NET_i_* > 0 (*NET_i_* < 0), and variable *i* is currently a net risk spillover (bearer), with the following forms of expression:

(8) $$TO_{i}\left(H\right)=\sum_{i=1,i\neq j}^{N}{\tilde{\theta }}_{ji}\left(H\right)$$

(9) $$FROM_{i}\left(H\right)=\sum_{i=1,i\neq j}^{N}{\tilde{\theta }}_{ij}\left(H\right)$$

(10) $$NET_{i}\left(H\right)=TO_{i}\left(H\right)-FROM_{i}\left(H\right)$$

Furthermore, the total connectedness index (*TCI*) indicates the total connectedness level between the cryptocurrency and energy markets. The degree of tail risk spillover and the linkage between the cryptocurrency and energy market tail risk contagion are positively correlated with the total connectedness index (*TCI*).

(11) $$TCI\left(H\right)=N^{-1}\sum_{i=1}^{N}TO_{i}\left(H\right)=N^{-1}\sum_{i=1}^{N}FROM_{i}\left(H\right)$$

Through the spectrum representation of variance decomposition, the spillover index in the time domain is divided into different frequency bands based on the quantile time domain. We can examine the tail risk spillover of cryptocurrency and China’s energy market from short-, medium-, and long-term perspectives based on various conditional quantiles, which are capable of accurately defining cyclical features and dynamic variation under various market situations. Consequently, the Fourier transform of QVMA(∞) may be employed to describe the spectral density of *x_t_* at frequency *ω*:

(12) $$S_{x}\left(\omega \right)=\sum_{h=-\infty }^{\infty }E\left(x_{t}x_{t-h}^{\prime }\right)e^{-i\omega h}=\Psi \left(e^{-i\omega h}\right)\sum_{t}\Psi^{\prime }\left(e^{+i\omega h}\right)$$

The frequency GFEVD, according to Chatziantoniou et al. [55], combines the GFEVD with the spectral density. Under the conditional quantile *τ*, *θ_ij_* (*ω*) reveals the portion of the spectrum of variable *i* affected by variable *j* and a further standardization process gives Formula (14):

(13) $$\theta_{ij}\left(\omega \right)=\frac{{\left(\sum \left(\tau \right)\right)}_{jj}^{-1}|\sum_{h=0}^{\infty }(\Psi \left(\tau \right)\left(e^{-i\omega h}\right)\sum \left(\tau \right){)}_{ij}{|}^{2}}{\sum_{h=0}^{\infty }(\Psi \left(e^{-i\omega h}\right)\sum \left(\tau \right)\Psi \left(\tau \right)\left(e^{i\omega h}\right){)}_{ij}}$$

(14) $${\tilde{\theta }}_{ij}\left(\omega \right)=\frac{\theta_{ij}\left(\omega \right)}{\sum_{k=1}^{N}\theta_{ij}\left(\omega \right)}$$

This study establishes a frequency band *d* = (*a*, *b*): *a*, *b* ∈ (−π,π), *a* < *b*, and analyzes the spillover level of variable *j* on variable *i* in a particular frequency band *d*. The goal is to explore the tail risk spillover between cryptocurrency and China’s energy market in various frequency domains. In order to examine the tail risk spillover between cryptocurrency and China’s energy market in three terms, this research splits frequency bands into three separate areas. Of these, the high-frequency band, *d* = (π/5, π), represents the short term of 1–5 days; the medium-frequency band, *d* = (π/20, π/5), represents the medium period of 5–20 days; and the low-frequency band, *d* = (0, π/20), represents the long term of more than 20 days. Thus, we can obtain the spillover index (*TO_i_*), spillover-in index (*FROM_i_*), net spillover index (*NETi*), and total connectedness index (*TCI*) in a particular frequency domain at various quantiles, and then the role of the entire network and the variables in it in risk contagion can be analyzed. The specific formulas are shown as follows:

(15) $$TO_{i}\left(d\right)=\sum_{i=1,i\neq j}^{N}{\tilde{\theta }}_{ji}\left(d\right)$$

(16) $$FROM_{i}\left(d\right)=\sum_{i=1,i\neq j}^{N}{\tilde{\theta }}_{ij}\left(d\right)$$

(17) $$NET_{i}\left(d\right)=TO_{i}\left(d\right)-FROM_{i}\left(d\right)$$

(18) $$TCI\left(d\right)=N^{-1}\sum_{i=1}^{N}TO_{i}\left(d\right)=N^{-1}\sum_{i=1}^{N}FROM_{i}\left(d\right)$$

### 3.3. Network Influencing Factors of Tail Risk Spillover

After using the QVAR model to measure the tail risk spillover effect of cryptocurrency and China’s energy market, this paper further studied the risk spillover influencing factors of the energy market by calculating the network topology index of China’s energy market taking into account cryptocurrency factors. Risk spillover can be understood as a form of “proximity” or “connection”. Its data breaks through the limitations of traditional geographical distance and produces a certain spatial agglomeration form. Therefore, we use economic distance to construct a spatial weight matrix and establish a spatial econometric model for subsequent research. Compared with the traditional geographical weight matrix that relies on simple rules set by humans, this weight matrix establishment method can better reflect the economic connection between variables and better capture the structure and asymmetric spillover of complex networks. However, the complexity of the risk spillover matrix also means that not all spatial econometric models can adapt. The spatial Durbin model considers both the spatial connection of the explained variables and the spatial connection of the explanatory variables. We choose it to establish a spatial econometric model for subsequent analysis.

According to Formula (14), we calculated the portion of the spectrum of variable *i* affected by variable *j*($\tilde{\theta }$_ij_ (ω)). We regard it as the weight of variable i on variable *j*. The one-way risk spillover effect between each two listed energy companies can be used as the spatial weight. According to the empirical analysis of the quantile time–frequency spillover framework, we selected the short-term (high frequency), medium-term (medium frequency), and long-term (low frequency) risk spillover matrices of the 0.50 conditional quantile (under normal state) with the largest time-varying volatility to obtain the directed spatial weight matrix under different time periods. Since the spillover index is not affected by the spillover-in index, it can more intuitively express the tail risk contagion intensity than the net spillover index. As a result, this paper selects the three-term spillover indexes under the normal risk condition (conditional median) calculated above as the explained variables. The relative outdegree is selected as the core explanatory variable to analyze the impact of the network connection on the spillover index. In addition, the following indicators are chosen as control variables in this paper. In terms of micro, the asset-liability ratio is utilized to measure the debt repayment ability of the company; the proportion of institutional investors’ holdings is employed to measure the development ability of the company; the total asset turnover rate is used to measure the operating ability of the company; and the rate of returns on common stockholders’ equity is employed to measure the profitability of the company. In terms of macro, this study selects the growth rate of total imports and exports of the company’s location to measure the foreign trade capacity. The regional GDP growth rate is selected to measure the level of economic development. On this basis, this paper constructs the spatial Durbin model with controlled individual effects for regression analysis. The specific regression model is as follows:

(19) $$\begin{array}{l} y_{i,t}=\delta \overset{N}{\underset{j=1}{\sum }}w_{i,j}y_{j,t}+\alpha_{1}Outdegree_{i,t}+\alpha_{2}\sum Controls_{i,t}\\ +\beta_{1}\overset{N}{\underset{j=1}{\sum }}w_{i,j}Outdegree_{i,t}+\beta_{2}\overset{N}{\underset{j=1}{\sum }}w_{i,j}\sum Controls_{i,t}+Id+\varepsilon_{i,t} \end{array}$$

where *i* and *j* represent each listed energy company; *t* represents the year; *N* represents the number of companies; *y_i,t_* represents the explained variable; *Outdegree_i_*_,*t*_ represents the core explanatory variable; *Controls_i_*_,*t*_ represents the set of control variables; *δ* measures the spatial spillover effect; *α* measures each variable’s effect on the company’s tail risk spillover, and *β* represents the spatial spillover effect of each variable; *w_ij_* represents the spatial weight matrix, including three types of spillover matrices: short-, medium- and long-term; *Id* represents the individual fixed effect; *ε* represents the random disturbance term.

## 4. Empirical Analysis

### 4.1. Data

Based on the industry categorization of the CSI Index, this paper selects 16 listed energy companies, excluding ST, delisted, and insufficient data companies. The specific samples are shown in Table 1. The total market value of the selected companies accounts for about 75% of the overall market value of the energy industry, which is representative of China’s energy market. For the selection of cryptocurrency variables, this paper uses Bitcoin (BTC) as a representative variable. Bitcoin was born in 2009 and applied blockchain technology to practice for the first time. This revolutionary design laid the underlying logic for thousands of subsequent cryptocurrencies. From its market position, Bitcoin occupies an absolute dominant position with a market share of 61.4%. In addition, institutional entry and regulatory discussions in mainstream countries are mostly centered on Bitcoin. Its algorithm setting of a constant total amount also makes Bitcoin known as “digital gold” and recognized by authoritative people. Bitcoin has the advantages of a large market share, strong influence, early issuance time, and complete data. In addition, before the ban on cryptocurrencies was issued, China contributed 65% of the world’s Bitcoin computing power. Therefore, this article selects Bitcoin as a representative variable of cryptocurrency for subsequent analysis. The sample time span ranges from 20 January 2014 to 19 January 2024, covering the stock market crash caused by monetary and credit easing and the stock crash caused by a large amount of leveraged funds in 2015, the stock market low in 2018, and the outbreak of COVID-19. The data on listed energy companies come from the CSMAR database, and the Bitcoin data come from https://cn.investing.com/ (accessed on 30 January 2024).

In order to ensure the coordination of the data, this paper uses the daily closing price as the basic data for listed energy companies and Bitcoin to obtain the logarithmic return rate, and the descriptive statistics of each variable are shown in Appendix A. Appendix A demonstrates that the skewness of the returns of the companies and Bitcoin is not 0. Additionally, Bitcoin and 13 companies have kurtosis greater than 3, and the others are close to 3. At the 1% significance level, the J-B statistical values of every variable are significantly higher than the critical value, rejecting the assumption of a normal distribution of the sequence. Hence, the ARMA–EGARCH–JSU model is employed to extract the volatility of each variable for subsequent analysis.

### 4.2. The Static Spillover Effect of Tail Risk in Cryptocurrency and Energy Market

**Figure 1:**
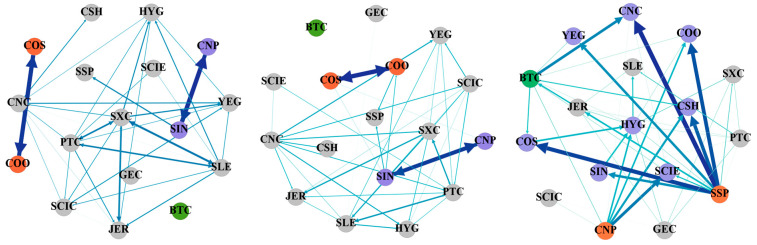
Time–domain tail risk spillover network between the cryptocurrency and energy markets.

**Figure 2:**
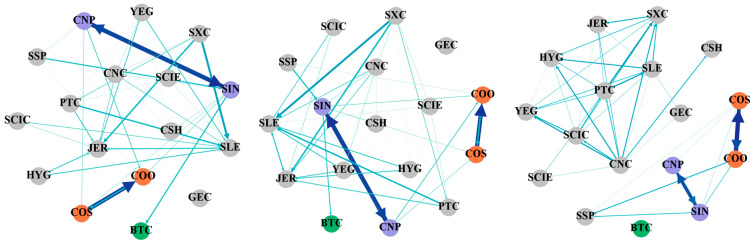
Frequency–domain tail risk spillover network between cryptocurrency and energy market. (conditional median).

**Figure 3:**
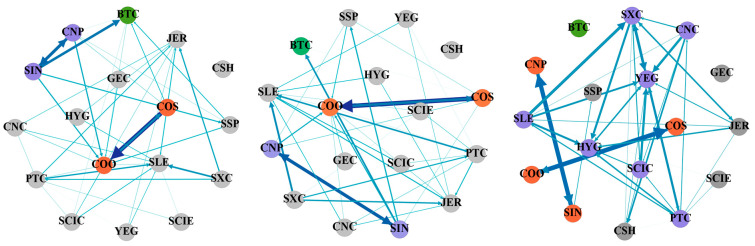
Frequency–domain tail risk spillover network between the cryptocurrency and energy markets (0.05 conditional quantile).

**Figure 4:**
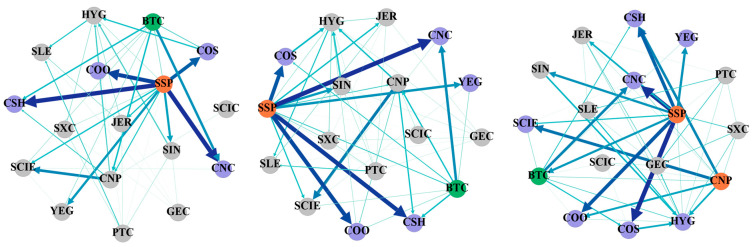
Frequency–domain tail risk spillover network between the cryptocurrency and energy markets (0.95 conditional quantile).

Utilizing the GFEVD of the leptokurtic QVAR model, we derive the tail risk static spillover matrix of Bitcoin and energy companies from the time–frequency dual perspective. Subsequently, the tail risk contagion network is constructed using the complex network approach. Considering the analysis of its topological structure, the paper explores the tail risk contagion impact of cryptocurrency and China’s energy market. Table 2, Table 3, Table 4 and Table 5 demonstrate the static spillover effect between Bitcoin and Chinese energy companies under different conditional quantiles in the time–frequency perspective. In the table, the forecast error variance is represented by the SELF column, which shows the contribution of the individual variable, he FROM column represents the tail risk spillover-in index, the TO column represents the tail risk spillover index, the NET column represents the net spillover index, and the TCI column represents the total connectedness index. The tail risk contagion network is shown in Figure 1, Figure 2, Figure 3 and Figure 4 from the standpoint of the frequency and time domain. The volatility spillover linkages between listed energy companies and Bitcoin are depicted as edges in this research. The direction of the arrows indicates the direction of risk spillover, while the edge colors—which vary from dark to light—represent the weight of the edges from high to low. For the convenience of analysis, this paper filters the links, retains only the top 50 links for subsequent analysis, and colors the nodes of key companies and Bitcoin so that readers can get a more intuitive understanding.

Table 2 depicts the time–domain static spillover effects of tail risks under various conditional quantiles of cryptocurrency and China’s energy market. Primarily, seen from the individual variable’s position within the entire risk network, CNC has the strongest risk spillover (113.11%) and the strongest net risk spillover (30.8%) under normal market conditions. YEG is the largest risk bearer (84.36%). At this time, Bitcoin’s impact on itself is as high as 84.77%, while the spillover and spillover-in effects are small. It illustrates that the connection between cryptocurrency and the energy market is not very close under normal market conditions. Compared with the normal state, SSP becomes the strongest risk spillover (306.76%) and net risk spillover (227.04%) under the extreme rising state. However, its spillover-in effect is smaller than other variables. It indicates that although this company has a small market share, its business influence is wide. Once it is hit, the amplitude of the entire market will also be large. In addition, HYG (137.88%), Bitcoin (136.42%), CNP (131.31%), and PTC (107.22%) are also strong risk spillovers, and their net spillover effects are all greater than 100%. SCIE (99.9%), HYG (99.86%), and COS (99.46%) are the main risk bearers. At this point, it can be seen that when the risk rises extremely, cryptocurrency, as an important risk spillover, will significantly affect China’s energy market. The spillover levels of various companies under the extreme decline state are similar to those in the normal state, while only the risk spillover-in level of Bitcoin has increased significantly (55.34%). This means that there is already a close connection between cryptocurrency and the energy market in the stage of risk accumulation.

Secondly, the total connectedness index under normal market conditions is 73.89%, which is lower than 82.66% and 94.83% under extreme decline conditions and extreme rise conditions, respectively. This suggests that the extreme conditions will cause the tail risk spillover effect to be underestimated by the spillover index under the 0.50 conditional quantile. Additionally, the self-effect to the forecast error variance under the 0.05 conditional quantile is 13.15% to 44.66%, while the self-effect under the 0.95 conditional quantile is 0.1% to 20.28%, both of which are smaller than the self-effect range of 15.64% to 84.77% under the normal state (0.50 conditional quantile). It shows that under extreme risk changes, the self-hysteresis effect of Bitcoin and various listed energy companies will decrease. Observing other data, the risk spillover-in effect under normal market conditions is the lowest, at 15.23%, and the highest is 84.36%, which is almost lower than the 55.34% to 86.85% under extreme decline condition and the 79.72% to 99.9% under extreme rise condition. As for the spillover effect, under an extreme decline state, the indexes of Bitcoin and listed energy companies have increased. Under the extreme rise condition, the risk spillover of 7 listed energy companies has increased significantly, and even the spillover index of Bitcoin has increased severalfold. This shows that under extreme risk conditions, the influence of cryptocurrency on the energy market will increase sharply. Once an extreme event occurs, cryptocurrency will also have a big effect on China’s energy market. In addition, the connections among companies in China’s energy market will become closer, and the impact from other companies in the same industry and the volatility spillover effects on other companies will increase accordingly. It will be easier for risks to spread throughout the energy market, and the impact on a certain company will affect other companies related to it. In summary, under extreme rising conditions, cryptocurrencies are more closely connected with the tail risks of companies in China’s energy market. This connectedness may become a source of systemic risk and financial instability. The spillover and contagion of financial risks of some companies are typical characteristics of systemic risks. Once a negative market shock occurs, the risk is likely to spread rapidly from an individual to the entire market, bringing challenges and uncertainties to financial supervision.

Figure 1 displays the network of the tail risk spillover effect. It shows the network contagion effects for extreme decline, normal, and extreme rise conditions sequentially from left to right. Overall, the extreme rise state has stronger risk aggregation and link strength, followed by the extreme decline state, and the normal state has the lowest aggregation and risk connectedness. Specifically, under the 0.05 and 0.50 conditional quantiles, COS and COO and CNP and SIN have strong two-way risk contagion. This is because COS and COO are both merged from professional companies of China National Offshore Oil Corporation, and their main businesses and corporate nature are homogeneous. As companies with many businesses at home and abroad, CNP and SIN are also closely connected. CNP mainly engages in oil production and refining, while SIN mainly engages in refining and produces downstream products. The crude oil source of CNP is mainly domestic mining, while SIN mainly imports crude oil from abroad. The business exchanges between them are also the main source of two-way risk contagion. Under extreme rise conditions, SSP shows a significant strong spillover effect. It can be seen that CSH, CNC, YEG, COS, and COO are all important risk bearers of SSP. In addition, as a leading company in the industry, CNP has a significant spillover effect on HYG, CSH, COO, SCIE, and other companies. This shows that companies of different sizes may become important sources of risk contagion in the industry and should be given equal attention. However, the strength of the connection of Bitcoin in normal and extreme decline states is low, and it is not shown in the top 50 strength connections. The degree of risk contagion increases under extreme conditions. This shows that risks do not spread easily between cryptocurrencies and China’s energy market in normal and extreme risk decline states, but when the overall market risk enters an extreme rise, the connection between cryptocurrencies and the energy market will increase significantly. Therefore, relevant departments need to be vigilant about the cross-market spread of tail risks caused by extreme events.

Based on the previous analysis, in comparison to normal market conditions, the total connectedness in extreme market conditions is amplified based on the previously mentioned analysis of the static spillover effect data from the time domain. The tail risk spillover impact under each extreme state is not well captured by the measurement of the spillover index under normal conditions, and the role of each variable will also change under different risk states. In addition, due to the volatility of the economic cycle, risk shocks produce responses of various frequencies during the propagation process, and there are high-frequency and low-frequency spillover effects. While low-frequency spillover effects are linked to changes in basic information, such as macroeconomic policy, high-frequency spillover effects can have an immediate influence on the market. High-frequency spillover effects have greater uncertainty and last longer, and they raise systemic risk contagion in the long term. Under distinct economic cycles, the tail risk contagion of different frequencies cannot be taken into account by time domain analysis. As a result, this paper presents a frequency domain examination of tail risk contagion between cryptocurrency and China’s energy market.

Table 3, Table 4 and Table 5 show the static spillover tables of tail risks between cryptocurrency and China’s energy market, using various conditional quantiles from a frequency domain perspective. Figure 2, Figure 3 and Figure 4 display the tail risk spillover network constructed on this basis. For ease of calculation, this paper categorizes 1–5 days as short-term, 5–20 days as medium-term, and over 20 days as long-term to examine the tail risk contagion in the cryptocurrency and China’s energy markets.

From the perspective of the frequency domain, Table 3 depicts the static spillover effect of tail risks between cryptocurrency and China’s energy market in a normal situation. First of all, we can see that the short-term total connectedness index is 2.06%, the medium-term total connectedness index is 5.77%, and the long-term total connectedness index is 66.06%. The degree of spillover over the long term surpasses that of the short and medium terms by a large margin. It is evident that under normal conditions, tail risk spillovers between cryptocurrency and China’s energy market are primarily driven by long-term risk spillovers. To be specific, in the short and medium term, we can see that SXC is the strongest risk spillover (3%, 8.35%), while SIN is the largest risk bearer (3.62%, 9.94%). In the long run, the risk spillover and spillover-in effects of listed energy companies are significantly different. YEG (78.77%) became the largest risk bearer, and CNC (102.76%) was the strongest risk spillover. This demonstrates that, under typical circumstances, the spillover index exhibits the same characteristics in the short- and medium-term, while the long-term risk spillover of China’s energy market will clearly display heterogeneity. Finally, observing the net tail risk spillover index, YEG exhibits the greatest net risk spillover in both the short and medium term (1.26%, 3.39%), indicating that it acts as the major risk spillover. COO exhibits the lowest net risk spillover (−1.46%, −3.89%) and acts as the main risk bearer. In the long term, YEG displays a negative net spillover index (−17.15%), and COO exhibits a positive net spillover index (13.57%), and the roles of the two are reversed. Moreover, CNC (28.49%) became the largest net risk spillover, and SCIE (−20.05%) became the largest risk bearer. This reveals that the tail risk structure of China’s energy market has undergone major changes in the long term, and the degree of risk spillover has increased significantly. Bitcoin becomes the net risk bearer in every cycle, indicating that cryptocurrency is affected by the volatility of energy companies under normal circumstances.

Figure 2 displays the tail risk spillover network from left to right for the short, medium, and long term. The depth of the edge color represents the weight of the edge, reflecting the risk spillover relationship between various energy companies and Bitcoin. It can be seen that the significant two-way influencing relationships between CNP and SIN, and COS and COO, mentioned in the time domain analysis, are still very obvious in different frequency domains, which indicates that the impact of business transactions and corporate structure affects each period. In addition, the relationship between Bitcoin and other nodes also changes with the cycle, but overall, the risk connection intensity between Bitcoin and listed energy companies in each time period under normal conditions is low. Therefore, under normal conditions, relevant departments should focus on risk prevention and control within the energy market, and, on this basis, increase the normalization of the monitoring of cryptocurrencies.

Table 4 describes the tail risk static spillover effect between cryptocurrency and China’s energy market based on the frequency–domain 0.05 conditional quantile. Firstly, from the perspective of the total connectedness index, the long-term total connectedness index is 59.26%, while the short-term is 6.56% and the medium-term is 16.84%, meaning that the tail risk of cryptocurrency and China’s energy market is still mainly determined by long-term factors under the extreme decline situation. Compared with the total connectedness index in the normal state, the short- and medium-term total spillover levels have increased, while the long-term spillover level has decreased. It indicates that the short- and medium-term impact capabilities have increased under the condition of extreme risk decline. Furthermore, the spillover and spillover-in indexes have increased in the short term and medium term compared with the conditional median, but the long-term index has decreased, which has the same trend as the total connectedness index. Finally, from a standpoint of net spillover, Bitcoin becomes the largest net risk bearer in the entire network under different economic cycles. YEG is the largest net risk spillover in the short term and medium term (3.23%, 7.65%). In the long run, SLE is the one with the largest net risk spillover (17.12%), which means that the positions of cryptocurrency and various listed energy companies in China’s energy market change accordingly in different periods.

Figure 3 displays the network in an extreme decline state based on the frequency domain viewpoint. Compared with the conditional median state, the edge strength in the three periods has deepened, and the clustering is more obvious, which is consistent with the results of time domain analysis. As a risk bearer, Bitcoin’s risk tolerance intensity has increased compared with the normal state. In the short and medium term, the risk spillover effect between CNP and SIN and COS and COO is obvious. In the long term, multiple nodes show significant spillover effects. The connection between regional listed energy companies such as YEG, SXC, HYG, PTC, SLE, CNC, and SCIC is closer. Therefore, under the extreme decline of risk, regulators should pay attention to the changes in the intensity of tail risk spillover under different economic cycles and focus on the risk contagion between regional companies.

From a frequency domain viewpoint, Table 5 illustrates the tail risk static spillover between cryptocurrency and China’s energy market under extreme upside situations. The current total connectedness indexes are 14.04%, 31.53%, and 49.27% for the short, medium, and long terms, respectively. The tail risk spillover between cryptocurrency and China’s energy market has increased in the extreme upside situation compared with the normal situation. The short- and medium-term risk contagion has significantly increased, while the long-term risk contagion has significantly decreased. Overall, medium- and long-term factors dominate the contagion of tail risks from cryptocurrency and China’s energy market amid extremely rising risks. Furthermore, in terms of the individual spillover index, the spillover level of SSP is significantly higher than that of all other individuals, indicating that China’s energy market is extremely vulnerable to emergencies at this node in the condition of extreme rise. The risk net spillover index also shows similar characteristics. When the risk of Bitcoin increases sharply, the net spillover index is positive, which is in contrast to the risk accumulation stage mentioned above. This means that when extreme events related to cryptocurrency occur, risks are transmitted from the cryptocurrency market to the energy market. Consequently, more attention should be paid to preventing the risk spread of Bitcoin and other cryptocurrencies to the energy market.

Figure 4 displays the network between cryptocurrency and China’s energy market under an extreme upside situation from a frequency domain perspective. Compared with the spillover network in the normal condition and the condition of extreme risk decline, the tail risk spillover in the condition of extreme risk increases greatly. Primarily, the short- and medium-term risk connectedness is improved compared with the normal situation and the extreme risk decline situation, while the long-term risk connectedness is weakened. Secondly, it can be seen from the figure that CNP shows a strong risk spillover effect on some companies in the long term. Regardless of the short, medium, or long term, Shanghai Petrochemical occupies an obvious middle position in the network diagram and has a strong risk spillover effect on CSH, CNC, COS, COO, and YEG. At the same time, it also affects other companies throughout the network. This also shows that in the process of risk prevention, we cannot just focus on industry-leading companies. Companies with smaller market capitalization may also play important roles in the entire network. Once a shock occurs, the risk contagion cannot be underestimated. The spillover effect of Bitcoin also gradually appears as the cycle lengthens, and it generates a more obvious spillover effect on companies with international business. Thus, relevant departments should not only be vigilant about the risk spillovers of leading companies but also identify the contagion of individual risks of individual energy companies with strong spillover effects. At the same time, they should also pay attention to the impact of volatility in the cryptocurrency market on listed energy companies.

By analyzing the tail risk spillovers between cryptocurrency and China’s energy market from a frequency domain perspective, we can understand that under different conditional quantiles, long-term factors dominate the tail risk spillovers between cryptocurrency and China’s energy market. In contrast to the normal situation, the total spillover levels in the short and medium term under extreme risk conditions have increased, while in the long term, they have decreased. The tail risk contagion effects between cryptocurrency and China’s energy market under different periods are heterogeneous, and their connections gradually increase as the period lengthens. Consequently, the tail risk contagion between cryptocurrency and China’s energy market should be accurately monitored under various risk conditions and various periods, illuminating the position of each individual in the risk network.

### 4.3. Dynamic Spillover Effect of Tail Risk Between Cryptocurrency and Energy Market

After analyzing the tail risk static spillover between cryptocurrency and China’s energy market, the tail risk dynamic spillover is investigated by the rolling window analysis method. Just as stated in the paper from Chatziantoniou et al. [55], the rolling window is set to 200. From both the time and frequency domain viewpoints, this article examines how the total tail risk spillover impact varies between cryptocurrency and China’s energy market under various market risk situations. Then, we demonstrate the dynamic variation of the net spillover effect of each variable, which provides new ideas for preventing systemic risks between cryptocurrency and China’s energy market under extreme conditions.

#### 4.3.1. Total Spillover Effect of Tail Risk Between Cryptocurrency and Energy Market

**Figure 5:**
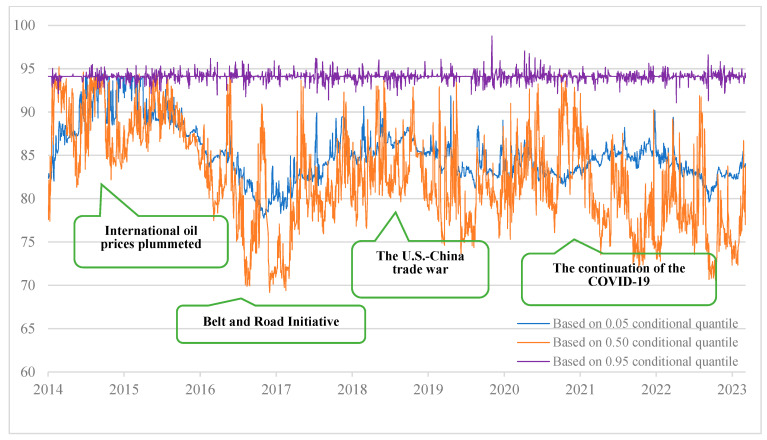
Time–domain tail risk total spillover effect between the cryptocurrency and energy markets.

We measure the total connectedness index of tail risk between cryptocurrency and China’s energy market based on 0.05, 0.50, and 0.95 conditional quantiles, respectively, to examine the total spillover of tail risk under extreme risk decline, normal state, and extreme risk increase. Compared with the traditional method of using conditional mean estimation, quantile regression can avoid the impact of outliers, and the results obtained are more robust to outliers. Figure 5 is measured from the time domain viewpoint. Furthermore, the total risk spillover effect of cryptocurrency and China’s energy market under the extremely rising state showed obvious heterogeneity with low volatility. The total risk spillover index of the extreme decline state and the normal state mainly ranged from 69% to 96%, with significant data volatility but values lower than those in the extreme rising state. Furthermore, time-varying features may be observed in the dynamic modification of the tail risk spillover index of China’s energy market and cryptocurrency under various conditional quantiles.

Taking the largest change in the total spillover effect under the normal condition as an example, global crude oil prices began to fall sharply in the second half of 2014. China is one of the world’s biggest net oil importers, so the volatility of global crude oil prices directly affects China’s energy import costs and energy prices. In the first half of 2015, China’s stock market experienced a wave of continuous rise, but then it began to fluctuate violently in June. On 24 August 2015, China’s stock market ushered in a crash known as “Black Monday”. This crash further exacerbated the market’s anxiety and had a negative impact on the entire economy and various industries, including the energy sector. Therefore, the total spillover level of tail risk was at a high level from 2014 to mid-2016. Since then, the Chinese government has increased its efforts to eliminate industries with excess capacity, especially the steel and coal industries, reducing excess capacity and optimizing the energy supply and demand structure. It has increased its support for clean energy, including the development and utilization of new energy, reducing dependence on traditional energy and diminishing environmental pollution and energy security risks. In addition, China has actively promoted the “Belt and Road” initiative and strengthened energy cooperation and exchanges with neighboring and international markets. Through diversified energy supply channels, the risks brought by individual supply sources are reduced, so the total risk spillover level in this stage has declined significantly.

The outbreak of Sino–US trade friction in 2018 had a wide-ranging impact on the global market, and China’s energy market was strongly impacted. The trade friction led to a slowdown in global economic growth and a downward adjustment in crude oil demand expectations, which had an adverse impact on energy prices and the market performance of related companies. The tail risk total connectedness index rose sharply to the second-highest level. Furthermore, the outbreak of COVID-19 in China around 2020 caused various industries to stop production. Energy demand dropped sharply, and the energy sector stock market was also hit, so the high total spillover level continued until after 2022. Until 2023, the impact of the epidemic gradually decreased, and international oil prices stabilized. The risks in the energy sector also gradually declined during this period, and the total spillover level of cryptocurrency and energy markets gradually declined.

**Figure 6:**
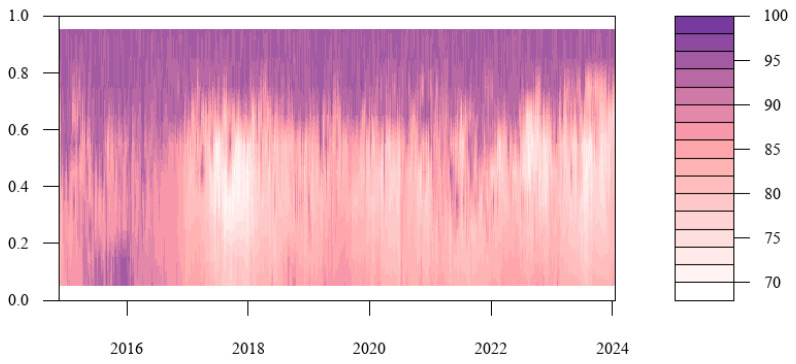
Quantile time–domain tail risk total spillover effect between the cryptocurrency and energy markets.

This paper extends the research to the calculation of the tail risk spillover index under different quantiles in order to further investigate the tail risk spillover level of cryptocurrency and China’s energy market under various shock scales. This allows us to show the tail risk spillover dynamic variation under extreme market shock in addition to concentrating on the conclusions of the tail risk spillover effect at particular conditional quantiles. Figure 6 presents the findings. The strong total spillover impact in the figure is represented by purple, the weak total spillover effect by white, and the steady increase in the total spillover effect by pink in the center. The figure shows that the total spillover effect of tail risk under extreme conditions is notably stronger than that under normal conditions, which shows that the total connectedness index of tail risk under normal conditions will underestimate extreme market conditions. Moreover, the total connectedness index under the state of extreme risk increase is much higher than that under the extreme risk decline condition, explaining that the risk spillover level is asymmetric between the left tail and the right tail. Under the extreme risk increase condition, a sharp change in stock price will lead to a sharp rise in irrational market sentiment, and it will extend to the entire cryptocurrency and China’s energy market through the market connection network. Driven by the “herd effect”, the adverse impact of extreme events will cause the tail risks of the cryptocurrency and China’s energy markets to resonate, and the level of risk contagion across markets will increase, which is very likely to cause systemic risks.

**Figure 7:**
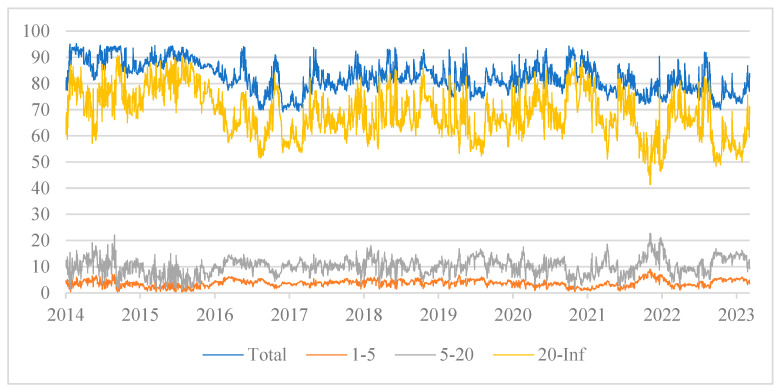
Frequency−domain tail risk total spillover effect between the cryptocurrency and energy markets (conditional median).

Additionally, this study uses a frequency domain approach to investigate the dynamic variation in the total spillover of tail risks between cryptocurrency and China’s energy market. It breaks down the total spillover impact into three cycles: short–, mid–, and long–term. The dynamic variations in the total tail risk spillover under the conditional median for each time horizon are displayed in Figure 7. Obviously, there is variation in the total tail risk spillover across various time periods. The short– and medium–term total tail risk spillovers first fall into a narrow range and do not entirely align with the overall curve’s trend. The short-term total connectedness index is below 10% with very low volatility, and the medium-term total connectedness index is below 30% with low volatility, indicating that short-term and medium-term shocks do not have a significant impact on the total tail risk spillover of cryptocurrency and China’s energy market. Then, the long-term total connectedness index is in a high range, and the volatility trends are nearly identical between the total curve and the long-term curve, which suggests that long–term factors dominate the total tail risk spillover effect of cryptocurrency and China’s energy market. Furthermore, although the risk spillover effect may not change significantly in the short term, as the horizon lengthens and the increasing uncertainty caused by shocks, the connection between Bitcoin and various energy companies will increase. This will broaden the scope of risk expansion and strengthen the intensity of contagion, resulting in a substantial rise in the long-term risk spillover effect between cryptocurrency and China’s energy market.

**Figure 8:**
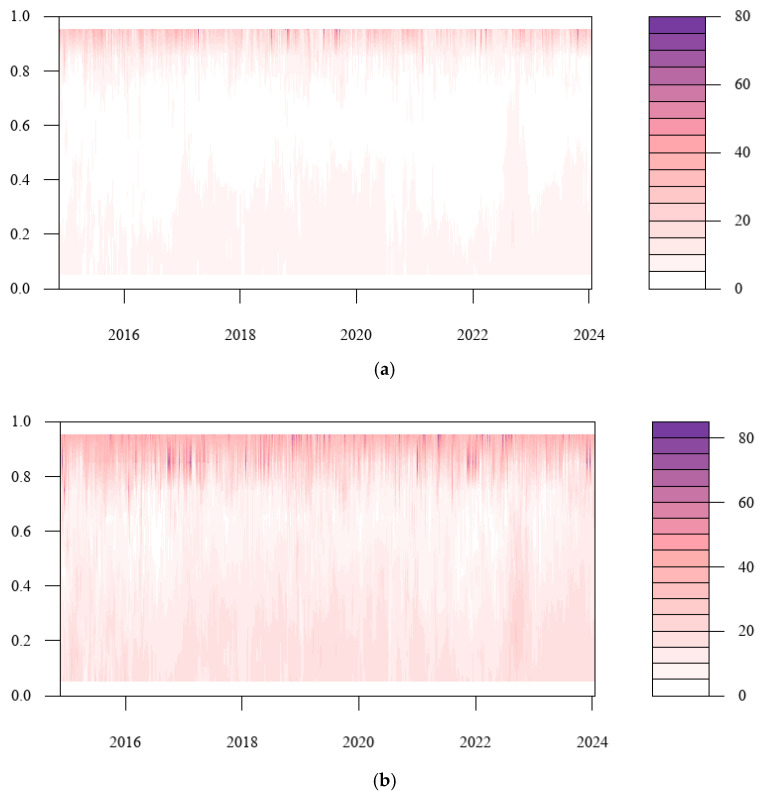

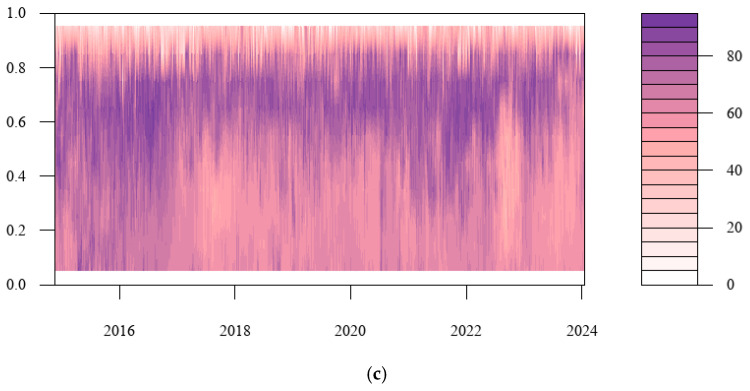
(**a**) Short–term tail risk total spillover effect between the cryptocurrency and energy markets under different quantiles. (**b**) Medium–term tail risk total spillover effect between the cryptocurrency and energy markets under different quantiles. (**c**) Long–term tail risk total spillover effect between the cryptocurrency and energy markets under different quantiles.

Based on the conditional median, we analyzed the total risk spillover from a frequency domain perspective under normal conditions. To further examine the spillover impact in various risk states, Figure 8a–c extend it to the dynamic variation of total risk spillover in each time horizon under various quantiles. As can be seen from the figure, the total tail risk spillover effect between cryptocurrency and China’s energy market increases significantly as the horizon grows. Primarily, in the short term, the tail risk spillover index is generally at a low level. In the medium term, risk spillovers are enhanced at high quantiles, while the long-term spillover effects are significantly enhanced at all quantiles. Hence, when extreme events occur in the market, investors should pay attention to the cross-contagion of tail risks brought about by long-term high volatility in cryptocurrency and energy markets. Additionally, the asymmetry of the tail increases with time, and the right tail spillover level was seen to be substantially greater than the left tail. This demonstrates that the total risk spillover effect is more obvious under the state of extreme risk increase. At this time, the risk linkage between Bitcoin and listed energy companies is stronger, and the extreme event shock is more likely to cause risk contagion. Consequently, regulatory authorities should formulate corresponding regulatory measures based on the heterogeneity of the total connectedness index under different risk states.

#### 4.3.2. The Net Spillover Effect of Tail Risks Between Cryptocurrency and China’s Energy Market

**Figure A1:**
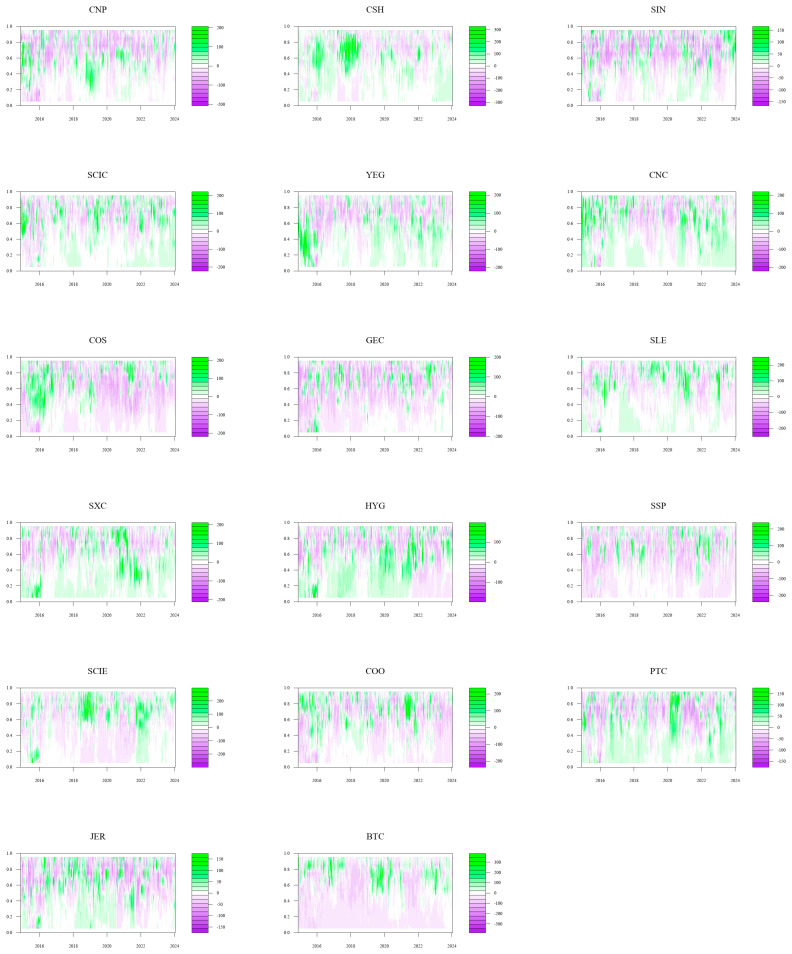
Time−domain tail risk net spillover effect between the cryptocurrency and energy markets.

This section examines the role of energy companies and Bitcoin in the overall network from a micro-individual perspective. This paper studies the individual spillover levels by displaying the dynamic variations of the net spillover impacts of Bitcoin and other energy firms from the time–frequency domain perspective. The company operates as a net risk spillover if the net spillover index is positive, which indicates that the spillover level is higher than the spillover-in level; if not, it functions as a net risk bearer. Due to space constraints, the specific results are presented in the Appendix. From a time domain viewpoint, Figure A1 shows that the tail risk’s net spillover impact is more pronounced at high quantiles, indicating that the net spillover effect of individuals becomes more obvious as the scale of the impact increases, which leads to increased volatility in the entire market. For cryptocurrency, we can see that Bitcoin is a net risk bearer in the low quantile, while it is a risk spillover in the high quantile. As for China’s energy market itself, companies with smaller market capitalizations such as GEC, SSP, and COS are in a situation where the net risk spillover is negative most of the time. This means that these companies tend to play the role of risk bearers under extreme shocks. As a result, smaller companies should prepare to deal with the crisis caused by risk contagion in daily business. However, the net risk spillover effect of large companies such as CNP and SIN is uncertain at high quantiles, indicating that when risks rise sharply, larger companies not only affect other companies in the market but also bear the risk. In addition, the likelihood of risk is also greatly increased. Furthermore, at the low quantile, CNC, SXC, HYG, and PTC are the main risk spillovers, indicating that under normal conditions and extreme decline conditions, there are still risk spillovers between companies. Through the analysis of net spillover effects in the time domain, it can be seen that the risk contagion ability of a company depends not only on the scale of the company but also on the geographic location of the company, the complexity of the customers, and the operating conditions. The regulatory authorities should attach importance to the support and risk monitoring of small and medium-sized companies and clarify the role of each company in the entire risk network at any time.

Then, we extend the time domain to the frequency domain and analyze the short-term, medium-term, and long-term net spillover effects. From the viewpoint of the changes in Bitcoin’s net spillover, it is a net risk bearer when the risk is low and a net risk spillover when the risk is high, which is consistent with previous research. It is clear from examining the net spillover impacts of different listed energy companies in China’s energy market that the net spillover effect in the time domain and the long-term net spillover effect are comparable. Furthermore, it is evident that long-term variables are principally responsible for the tail risk contagion between cryptocurrency and China’s energy market. The short- and medium-term graphs, however, exhibit comparable traits that differ from those of the long-term graphs. CSH, YEG, CNC, SXC, and HYG are the main net risk spillovers, and CNP, SIN, SLE, SSP, and COO are the net risk bearers. In short, investors and regulators should be wary of the cross-contagion of tail risks brought about by long-term risks. At the same time, when dealing with shocks of different degrees, directions, and periods, appropriate response measures should be formulated according to their differences.

### 4.4. Spatial Spillover Effects and Network Influencing Factors of Tail Risks in Energy Market

The complex network method provides a way to analyze connections from the spatial perspective. Through network topology indicators, the position and importance of energy companies in the network can be depicted. Therefore, this section combines spatial econometric models with complex network methods to analyze spatial spillover effects. Based on the above network risk analysis of cryptocurrency and China’s energy market, the spatial spillover effect of China’s energy market, considering cryptocurrency factors, was studied using the spillover matrix from a frequency domain perspective. Table 6 shows the test results of Moran’s I index using short-term (W1), medium-term (W2), and long-term (W3) risk spillover matrices for spatial autoregressive analysis. The data in the table reveal that there is a significant positive spatial connection in risk spillover effects from 2014 to 2023. After determining that the risk matrix has a spatial connection, the influence of the network connection on the tail risk spillover effects in various time horizons was further explored.

This paper uses the spatial Durbin model to measure the spatial spillover effects of tail risks in China’s energy market. In Table 7, W1 is the short-term (high-frequency) matrix, W2 is the mid-term (medium-frequency) matrix, and W3 is the long-term (low-frequency) matrix. The core explanatory variable selected is the relative outdegree, which refers to the ratio of the sum of the weights of the edges emanating from the node to the maximum degree. It measures the degree of spillover of each node in the network. The table makes it clear that the short– and medium–term outcomes produced by utilizing the W1 and W2 matrices are quite comparable. At the 1% level, the relative outdegree is significant and has a positive effect on the tail risk spillover level of China’s energy market. The asset–liability ratio has a positive effect on the spillover index, indicating that companies with high debt solvency have more extensive business and more obvious spillover effects. There is a negative relationship between the growth rate of total imports and exports of the company’s location and spillover effects, indicating that tightening foreign trade can inhibit risk spillovers to a certain extent. At the same time, the relative outdegree has a negative spatial spillover effect in both the short and medium term, indicating that network connectedness has a negative conductive effect on the spillover index. In other words, the spillover level of a certain company is negatively affected by the risk spillover of related companies. The spatial autoregression coefficient of the short-term matrix is 0.865, and the spatial autocorrelation coefficient of the medium-term matrix is 0.802, both of which are significant at the 1% level. This indicates that the tail risk spillover index of China’s energy market has a positive spatial spillover impact on itself.

The performance of long-term spatial econometric regression using the W3 matrix is slightly different. The long-term relative outdegree still has a positive impact on the spillover effect, but the coefficient is much higher than the short- and medium-term, indicating that the long-term network connection on tail risk spillover should be given more attention in risk management. The asset–liability ratio, the proportion of institutional investors’ holdings, the total asset turnover rate, and the growth rate of total imports and exports of the company’s location all have a positive spatial spillover effect on the spillover index. It indicates that these factors have a positive transmission effect on energy companies’ spillover effects in the long run. The rate of returns on common stockholders’ equity and the regional GDP growth rate have negative spatial spillover effects, that is, they play a negative transmission role. The long-term matrix’s spatial autoregression coefficient, which is 0.449 and significant at the 1% level, shows that each energy company’s spillover index will continue to have a positive spatial spillover impact on itself over the long run.

This paper further analyzes the direct, indirect, and total effects of each risk matrix in order to further analyze the spatial spillover effect of each period, as the coefficient of the spatial Durbin model cannot directly reflect the marginal effect of the corresponding explanatory variable on the explained variable. The influence of the variable on the energy company’s risk spillover effect is known as the direct effect, while the indirect effect is the impact of the relevant energy companies on the company. The sum of the direct and indirect effects is known as the total effect, and Table 8 displays all the outcomes. Evidently, the relative outdegree has both direct and indirect effects on the tail risk spillover effect of China’s energy market, indicating that the impact of the company on other companies and the outdegree of related companies will affect the change in the spillover index. The increase in the relative outdegree of the company leads to a positive change in the spillover index, while the increase in the relative outdegree of related companies leads to a negative change in the spillover index. Consequently, from the perspective of spatial connection, it is possible to examine the effect of network connectedness on the tail risk spillover index of China’s energy market. This paper explores the spatial risk connection relationship based on the previous use of methods such as the two-stage instrumental variable method and provides new evidence for the study of China’s energy market.

## 5. Conclusions and Suggestions

In the financial field, the rapid rise of the cryptocurrency market has attracted widespread attention. At the same time, the huge energy consumption behind it is concerning. The regulatory authorities are facing difficulty in preventing and controlling the tail risk contagion between the cryptocurrency and energy markets. To investigate the tail risk spillover impact between the cryptocurrency and energy markets, this study uses two research objects: Chinese listed energy businesses and Bitcoin, an important cryptocurrency. Considering the leptokurtosis and fat-tail properties of financial returns, this paper initially assumes that the variable returns follow the JSU distribution and then uses the stochastic volatility model with asymmetric features to determine the volatility. Subsequently, a static volatility spillover network of cryptocurrency and China’s energy market is constructed from a dual perspective of time and frequency using the GFEVD of QVAR, and its tail risk cross-contagion features are analyzed. Then, based on the quantile time–frequency spillover framework, the tail risk spillover dynamic variations under the time–frequency domain are further explored. Finally, we use the spatial Durbin model combined with the spillover matrix in various frequency domains to analyze the influencing factors of the spatial spillover effect in China’s energy market.

From the static spillover network of tail risk between cryptocurrency and China’s energy market, it is evident that the tail risk network is heterogeneous in the face of risk shocks of different scales and different periods. From the standpoint of the time domain, the risk spillover effect in extreme situations is more significant than that in the normal situation, and the spillover effect is most obvious when the market is in an extreme upward situation. Bitcoin mainly acts as a risk bearer in an extreme falling state, but it becomes a risk spillover in the extreme upward condition. Regarding the frequency domain viewpoint, the long-term strongest connectedness is demonstrated by the tail risk spillover effects under different market situations. In addition, under extreme market situations, some companies with smaller scales show strong risk spillover effects in different periods. Hence, policymakers should focus on the two-way contagion effect of tail risk between the cryptocurrency and energy markets under an extreme market state and formulate policies to focus on the spread of the tail spillover effect in the long run. In addition, regulatory authorities cannot judge the risk level of companies only from the perspective of company size; they must also conduct a comprehensive analysis in combination with other factors, such as their business structure and geographical location.

Furthermore, within a time–frequency dual framework, the leptokurtic QVAR-based time–frequency spillover methodology is better able to capture the dynamic variation of risk contagion between cryptocurrency and China’s energy market. There are distinct time-varying features to the total tail risk spillover impact in China’s energy market and cryptocurrency market. There are varying degrees of influence from extreme events. For example, the COVID-19 outbreak, trade tensions between China and the US, and the abrupt volatility in global oil prices have all had a major impact on the total spillover effect. At the same time, the total connectedness index of tail risk based on the normal state underestimates the risk level when the risk rises and falls extremely, and the risk spillover level exhibits asymmetry between the left tail and the right tail. From the frequency domain viewpoint, long-term factors are the major determinants of the total spillover effect of tail risk in cryptocurrency and China’s energy market. Hence, regulators should build a differentiated financial risk prevention mechanism, pay attention to the cyclical volatility of risk spillovers, and make timely adjustments to economic policies according to the trend of risk volatility.

Ultimately, the results of spatial econometric regression show that the relative outdegree has a significant positive effect on the spillover index of the company, while the relative outdegree of the associated companies has a significant negative effect on the spillover index of the company. Both effects are more obvious with an increase in the time horizon. In addition, the long-term asset–liability ratio, the proportion of institutional investors’ holdings, the total asset turnover rate, and the growth rate of total imports and exports of the company’s location all have a positive transmission effect on the spillover index, while the rate of returns on common stockholders’ equity and the regional GDP growth rate have a negative transmission effect. These conclusions show that network connectedness, financial indicators, and macroeconomic indicators all have an impact on the spillover index of listed energy companies. If the spatial spillover effect between companies is ignored in the regulatory process, it may affect the measurement of risk spillover effects.

Based on the above research conclusions, we put forward the following suggestions for monitoring the cryptocurrency market and risk prevention in China’s energy market. For the cryptocurrency market, China should always be vigilant about the impact of cryptocurrency price changes on cross-border business, especially cross-border business transactions with Chinese energy companies. For risk prevention and control in China’s energy market, policymakers should establish a dynamic and multi-level cross-market risk monitoring system. First, the monitoring system should establish a macro-risk monitoring mechanism to warn of major external events and jointly enhance the overall risk resistance of China’s energy market with regulatory agencies. Second, the system should establish a risk differentiation monitoring mechanism in the energy market at the individual company level. Regulators should focus on companies that participate in cross-border energy transactions or are at the center of the entire risk network. The specific evaluation metrics include business composition, financial metrics, and topological indicators such as the relative outdegree within complex networks. It is worth noting that regulatory authorities should pay special attention to the systemic importance of some small companies under extreme risk conditions to avoid the spread of systemic risks caused by neglect of small and medium-sized companies. In addition, the system should also establish a dynamic monitoring mechanism for risk contagion in the cryptocurrency and energy markets and monitor the cross-market risk contagion mechanisms in different time periods. Based on the conclusions, regulators should pay special attention to the impact of long-term factors on risk spillover effects. Finally, both investors and regulators should follow the trend of energy policy, actively promote the low-carbon transformation of energy companies, achieve business diversification, and increase the risk prevention and control level of China’s entire energy market from a micro perspective.

## Figures and Tables

**Table 1:** Sample selection table.

Code	Name	Variable	Code	Name	Variable
601857	China National Petroleum Corporation(Beijing, China)	CNP	601699	Shanxi Lu’an Environmental Energy Development Co., Ltd. (Changzhi, Shanxi, China)	SLE
601088	China Shenhua Energy Co., Ltd. (Beijing, China)	CSH	000983	Shanxi Coking Coal Group Co., Ltd. (Taiyuan, Shanxi, China)	SXC
600028	China Petroleum & Chemical Corporation (Beijing, China)	SIN	600348	Shan Xi Hua Yang Group New Energy Co., Ltd. (Yangquan, Shanxi, China)	HYG
601225	Shaanxi Coal Industry Co., Ltd. (Xi’an, Shaanxi, China)	SCIC	600688	Sinopec Shanghai Petrochemical Co., Ltd. (Shanghai, China)	SSP
600188	Yankuang Energy Group Co., Ltd.(Jining, Shandong, China)	YEG	600546	Shanxi Coal International Energy Group Co., Ltd. (Taiyuan, Shanxi, China)	SCIE
601898	China Coal Energy Co., Ltd. (Beijing, China)	CNC	600583	Offshore Oil Engineering Co., Ltd. (Tianjin, China)	COO
601808	China Oilfield Services Limited (Tianjin, China)	COS	601666	Pingdingshan Tianan Coal Mining Co., Ltd. (Pingdingshan, Henan, China)	PTC
600256	Guanghui Energy Co., Ltd. (Urumqi, Xinjiang, China)	GEC	000937	Jizhong Energy Resources Co., Ltd. (Xingtai, Hebei, China)	JER

**Table 2:** Time–domain tail risk static spillover between the cryptocurrency and energy markets.

	0.05 Conditional Quantile				0.50 Conditional Quantile				0.95 Conditional Quantile			
	SELF	FROM	TO	NET	SELF	FROM	TO	NET	SELF	FROM	TO	NET
TCI	82.66				73.89				94.83			
CNP	16.86	83.14	78.58	−4.56	25.9	74.1	77.61	3.52	9.39	90.61	131.31	40.7
CSH	17.81	82.19	73.68	−8.51	25.83	74.17	73.24	−0.93	1.17	98.83	52.88	−45.95
SIN	16.1	83.9	85.94	2.04	27.15	72.85	84.97	12.12	0.56	99.44	53.65	−45.79
SCIC	13.81	86.19	98.82	12.63	17.59	82.41	85.26	2.86	6.47	93.53	64.16	−29.36
YEG	14.69	85.31	89.18	3.86	15.64	84.36	71.88	−12.49	3.28	96.72	64.16	−32.56
CNC	13.35	86.65	100.19	13.54	17.69	82.31	113.11	30.8	0.71	99.29	60.21	−39.07
COS	16.82	83.18	78.57	−4.61	26.17	73.83	65.17	−8.65	0.54	99.46	48.73	−50.72
GEC	17.67	82.33	77.08	−5.24	25.51	74.49	69.33	−5.16	8.38	91.62	92.86	1.24
SLE	13.15	86.85	102.88	16.03	18.19	81.81	94.4	12.59	3.15	96.85	68.96	−27.89
SXC	13.23	86.77	101	14.23	17.05	82.95	91.91	8.95	7.47	92.53	76.26	−16.27
HYG	15.51	84.49	84.18	−0.32	17.67	82.33	68.11	−14.22	0.14	99.86	137.88	38.02
SSP	19.04	80.96	67.07	−13.89	27.52	72.48	58.54	−13.93	20.28	79.72	306.76	227.04
SCIE	16.95	83.05	78.47	−4.58	27.56	72.44	50.12	−22.31	0.1	99.9	73.33	−26.57
COO	17.38	82.62	76.83	−5.78	33.15	66.85	75.05	8.2	0.56	99.44	47.76	−51.68
PTC	13.22	86.78	103.28	16.51	17.77	82.23	96.96	14.73	9.94	90.06	107.22	17.17
JER	14.54	85.46	90.43	4.97	18.67	81.33	76.31	−5.03	7.84	92.16	89.6	−2.56
BTC	44.66	55.34	19.03	−36.31	84.77	15.23	4.19	−11.04	7.84	92.16	136.42	44.26

**Table 3:** Frequency–domain tail risk static spillover between the cryptocurrency and energy markets (conditional median).

	Short-Term				Medium-Term				Long-Term			
	SELF	FROM	TO	NET	SELF	FROM	TO	NET	SELF	FROM	TO	NET
TCI	2.06				5.77				66.06			
CNP	1.33	3.06	2.31	−0.75	3.61	8.41	6.36	−2.05	20.96	62.63	68.95	6.32
CSH	0.71	1.78	1.89	0.1	2	5	5.3	0.3	23.11	67.39	66.06	−1.33
SIN	1.42	3.62	2.62	−1	3.86	9.94	7.17	−2.77	21.87	59.29	75.19	15.9
SCIC	0.5	1.4	2.42	1.02	1.4	4.01	6.84	2.82	15.69	77	76	−1
YEG	0.47	1.45	2.71	1.26	1.34	4.15	7.54	3.39	13.82	78.77	61.61	−17.15
CNC	0.58	2.07	2.7	0.62	1.64	5.96	7.65	1.7	15.47	74.28	102.76	28.49
COS	0.92	1.45	1.88	0.43	2.6	4.33	5.25	0.92	22.65	68.04	58.05	−9.99
GEC	0.52	0.8	1.29	0.48	1.49	2.42	3.67	1.25	23.49	71.27	64.38	−6.89
SLE	0.91	3.24	2.52	−0.72	2.49	9	7.04	−1.96	14.79	69.57	84.83	15.26
SXC	0.5	1.84	3	1.16	1.38	5.13	8.35	3.22	15.17	75.98	80.54	4.57
HYG	0.6	1.6	2.3	0.7	1.68	4.48	6.39	1.91	15.39	76.26	59.4	−16.85
SSP	1.21	1.63	1.44	−0.2	3.32	4.85	4	−0.85	22.99	66	53.12	−12.88
SCIE	1.04	2.08	1.47	−0.61	2.86	5.75	4.09	−1.66	23.66	64.61	44.57	−20.05
COO	1.71	2.86	1.4	−1.46	4.64	7.99	4.1	−3.89	26.8	56	69.57	13.57
PTC	0.64	2.09	2.62	0.53	2.61	5.82	7.45	1.63	15.37	74.31	86.88	12.57
JER	0.96	3.01	2.35	−0.66	21.82	8.24	6.55	−1.69	15.1	70.08	67.4	−2.68
BTC	8.76	1.05	0.14	−0.91	84.77	2.66	0.39	−2.27	54.19	11.52	3.67	−7.86

**Table 4:** Frequency–domain tail risk static spillover between the cryptocurrency and energy markets (0.05 conditional quantile).

	Short-Term				Medium-Term				Long-Term			
	SELF	FROM	TO	NET	SELF	FROM	TO	NET	SELF	FROM	TO	NET
TCI	6.56				16.84				59.26			
CNP	1.52	7.44	6.51	−0.93	3.9	19.07	16.57	−2.5	11.44	56.64	55.5	−1.13
CSH	0.96	4.6	5.79	1.19	2.63	12.51	14.93	2.43	14.21	65.08	52.95	−12.13
SIN	1.55	8.11	7.22	−0.89	3.92	20.47	18.26	−2.21	10.63	55.32	60.46	5.14
SCIC	0.92	5.77	7.69	1.92	2.47	15.47	19.81	4.34	10.42	64.95	71.32	6.37
YEG	0.64	3.75	6.99	3.23	1.79	10.43	18.08	7.65	12.25	71.13	64.11	−7.02
CNC	0.87	5.56	7.78	2.21	2.36	15.14	20.06	4.91	10.12	65.95	72.35	6.41
COS	1.35	6.49	6.75	0.27	3.52	17.09	17.07	−0.02	11.95	59.61	54.75	−4.86
GEC	1.03	4.99	6.22	1.23	2.77	13.36	15.92	2.56	13.87	63.98	54.95	−9.03
SLE	1.24	8.17	7.81	−0.36	3.17	20.94	20.21	−0.73	8.74	57.74	74.86	17.12
SXC	0.83	5.42	7.85	2.43	2.25	14.68	20.27	5.59	10.16	66.66	72.88	6.21
HYG	0.8	4.35	6.59	2.24	2.19	11.95	17	5.05	12.51	68.2	60.59	−7.61
SSP	1.73	7.3	5.67	−1.63	4.43	18.69	14.32	−4.37	12.87	54.97	47.08	−7.89
SCIE	1.32	6.65	6.19	−0.47	3.47	17.39	15.89	−1.5	12.16	59	56.39	−2.61
COO	1.98	9.39	6.18	−3.21	4.8	22.78	15.85	−6.93	10.61	50.45	54.8	4.36
PTC	1.11	7.3	7.92	0.63	2.87	18.91	20.48	1.58	9.24	60.57	74.87	14.3
JER	1.29	7.55	6.82	−0.73	3.31	19.39	17.69	−1.71	9.93	58.52	65.92	7.41
BTC	7.2	8.66	1.51	−7.15	15.17	18.07	3.93	−14.14	22.29	28.61	13.59	−15.02

**Table 5:** Frequency–domain tail risk static spillover between cryptocurrency and China’s energy market (0.95 conditional quantile).

	Short-Term				Medium-Term				Long-Term			
	SELF	FROM	TO	NET	SELF	FROM	TO	NET	SELF	FROM	TO	NET
TCI	14.04				31.53				49.27			
CNP	0.86	9.67	16.06	6.39	2.32	24.89	38.99	14.1	6.07	56.19	70.03	13.84
CSH	0.01	15.53	7.46	−8.06	0.14	33.72	17.38	−16.33	0.73	49.88	27.84	−22.04
SIN	0.17	17.96	6.84	−11.12	0.32	37.33	16.77	−20.56	0.16	44.05	28.28	−15.77
SCIC	0.94	12.91	8.87	−4.04	2.23	31.07	20.49	−10.59	3.37	49.47	34.98	−14.49
YEG	0.65	16.7	8.7	−8.01	1.36	35.74	20.38	−15.35	1.39	44.16	34.9	−9.26
CNC	0.07	16.99	7.41	−9.59	0.17	35.58	18.18	−17.4	0.39	46.8	33.16	−13.64
COS	0.06	15.66	6.55	−9.11	0.14	33.44	15.6	−17.84	0.3	50.4	25.4	−24.99
GEC	0.89	10.24	14.63	4.39	2.29	25.67	32.09	6.41	5.14	55.76	47.16	−8.6
SLE	0.71	14.61	9.16	−5.45	1.36	31.94	21.67	−10.27	1.43	49.94	37.68	−12.26
SXC	1.2	14.12	10.42	−3.7	2.61	31.35	24.19	−7.16	3.75	46.97	41.72	−5.25
HYG	0	14.29	20.95	6.66	0	34.65	46.34	11.69	0.1	50.94	72.03	21.09
SSP	2.84	12.31	50.44	38.12	6.43	27.15	107.58	80.43	10.64	40.62	152.69	112.08
SCIE	0.03	15.22	11.93	−3.3	0.04	34.49	25.96	−8.53	0.05	50.16	36.34	−13.82
COO	0.09	17.14	6.74	−10.4	0.2	35.84	15.81	−20.03	0.23	46.5	24.66	−21.84
PTC	1.37	12.53	16.27	3.74	3.19	29.24	36.05	6.81	5.37	48.3	55.82	7.52
JER	1.3	14.61	12.91	−1.7	2.78	31.76	29.24	−2.52	3.84	45.71	48.06	2.34
BTC	0.73	8.15	23.32	15.17	1.97	22.23	49.37	27.14	5.2	61.71	66.82	5.11

**Table 6:** Moran’s I index of short-term, medium-term, and long-term spillover effects under normal conditions.

Year	Moran’s I (W1)	*z*	*p*-Value	Moran’s I (W2)	*z*	*p*-Value	Moran’s I (W3)	*z*	*p*-Value
2014	0.234	4.139	0.000	0.206	4.393	0.000	0.094	6.745	0.000
2015	0.267	3.976	0.000	0.255	4.429	0.000	0.112	6.598	0.000
2016	0.421	5.575	0.000	0.344	5.598	0.000	0.114	6.603	0.000
2017	0.321	4.465	0.000	0.281	4.687	0.000	0.122	9.138	0.000
2018	0.244	4.069	0.000	0.195	3.747	0.000	0.141	9.767	0.000
2019	0.332	4.761	0.000	0.300	5.100	0.000	0.158	8.248	0.000
2020	0.260	3.878	0.000	0.238	4.216	0.000	0.129	7.097	0.000
2021	0.310	4.348	0.000	0.216	3.834	0.000	0.099	6.294	0.000
2022	0.434	5.861	0.000	0.373	5.975	0.000	0.099	7.088	0.000
2023	0.393	5.327	0.000	0.304	5.103	0.000	0.114	6.751	0.000

**Table 7:** Spatial econometric model regression results.

Variable	W_1_	W_2_	W_3_
Outdegree	0.229 ***	0.617 ***	3.465 ***
	(0.005)	(0.014)	(0.090)
ALR	0.020 *	0.059 *	−0.189
	(0.011)	(0.033)	(0.220)
SHR	0.025 *	0.037	0.132
	(0.014)	(0.043)	(0.319)
ATR	0.007 **	0.012	0.200 ***
	(0.003)	(0.009)	(0.065)
ROE	−0.002	0.016	0.217
	(0.007)	(0.021)	(0.141)
GRnx	−0.011 ***	−0.027 **	0.061
	(0.004)	(0.011)	(0.079)
GRgdp	0.002	0.043	−0.168
	(0.010)	(0.030)	(0.241)
W × Outdegree	−0.218 ***	−0.611 ***	−3.603 ***
	(0.008)	(0.025)	(0.189)
W × ALR	−0.071 **	−0.279 ***	2.081 **
	(0.030)	(0.104)	(0.993)
W × SHR	−0.106 **	−0.166	6.939 ***
	(0.045)	(0.152)	(2.164)
W × ATR	−0.015 *	−0.015	1.402 ***
	(0.008)	(0.025)	(0.353)
W × ROE	0.001	−0.095	−2.344 ***
	(0.017)	(0.058)	(0.737)
W × GRnx	0.011	0.068 **	0.553 **
	(0.008)	(0.026)	(0.269)
W × GRgdp	−0.003	−0.105	−1.360 **
	(0.200)	(0.065)	(0.660)
ρ	0.865 ***	0.802 ***	0.449 ***
	(0.029)	(0.044)	(0.129)
$\sigma^{2}$	9.17 × 10^−6^ ***	0.0001 ***	0.007 ***
	(1.10 × 10^−6^)	(0.00001)	(0.001)
Individual fixed effects	Yes	Yes	Yes
N	160	160	160
$R^{2}$	0.915	0.894	0.826

Note: ***, **, and * indicate that the regression coefficient is significant at the 1%, 5%, and 10% levels, respectively; the standard errors are in parentheses, and *N* is the number of observations; the same below.

**Table 8:** Direct, indirect, and total effects of spillover effect factors in each period.

Variable	Direct			Indirect			Total		
	W_1_	W_2_	W_3_	W_1_	W_2_	W_3_	W_1_	W_2_	W_3_
Outdegree	0.215 ***	0.554 ***	3.048 ***	−0.129 ***	−0.524 ***	−3.309 ***	−0.085 *	0.031	−0.262
	(0.005)	(0.011)	(0.062)	(0.041)	(0.088)	(0.270)	(0.043)	(0.093)	(0.273)
ALR	−0.020 *	−0.065 **	0.223	−0.345 **	−1.014 ***	3.313 **	−0.364 **	−1.079 ***	3.537 **
	(0.012)	(0.030)	(0.169)	(0.141)	(0.337)	(1.344)	(0.150)	(0.358)	(1.398)
SHR	−0.038 *	−0.035	1.600 ***	−0.570 **	−0.615	11.558 ***	−0.608 **	−0.650	13.159 ***
	(0.019)	(0.049)	(0.348)	(0.240)	(0.584)	(4.036)	(0.257)	(0.624)	(4.308)
ATR	0.001	0.009	0.069	−0.060	−0.024	2.128 ***	−0.060	−0.015	2.197 ***
	(0.003)	(0.007)	(0.052)	(0.041)	(0.095)	(0.467)	(0.043)	(0.100)	(0.492)
ROE	−0.002	−0.027	−0.243 **	0.003	−0.352	−3.632 ***	−0.001	−0.379	−3.875 ***
	(0.007)	0.018	(0.120)	(0.091)	(0.221)	(1.336)	(0.097)	(0.233)	(1.404)
GRnx	−0.011 **	−0.002	0.183 **	0.005	0.204 **	0.938 *	−0.005	0.202 *	1.120 **
	(0.004)	(0.011)	(0.075)	(0.040)	(0.097)	(0.509)	(0.043)	(0.103)	(0.539)
GRgdp	0.001	0.005	−0.461 **	−0.005	−0.312	−2.322 **	0.005	−0.307	−2.783 **
	(0.012)	(0.032)	(0.227)	(0.105)	(0.251)	(1.137)	(0.113)	(0.268)	(1.198)

**Figure A2:**
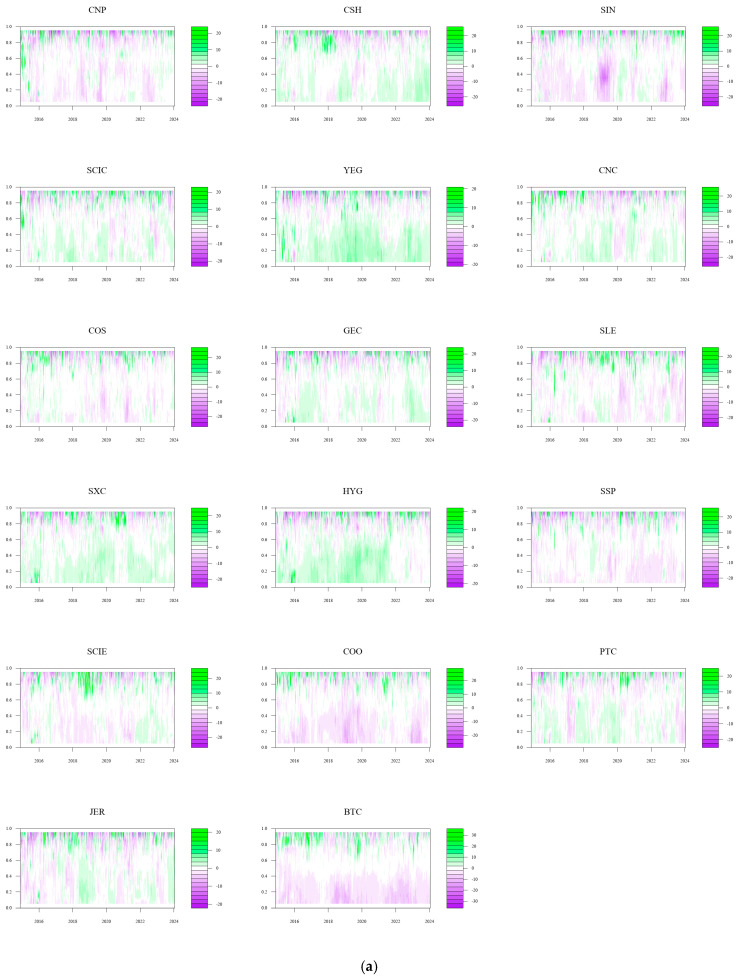

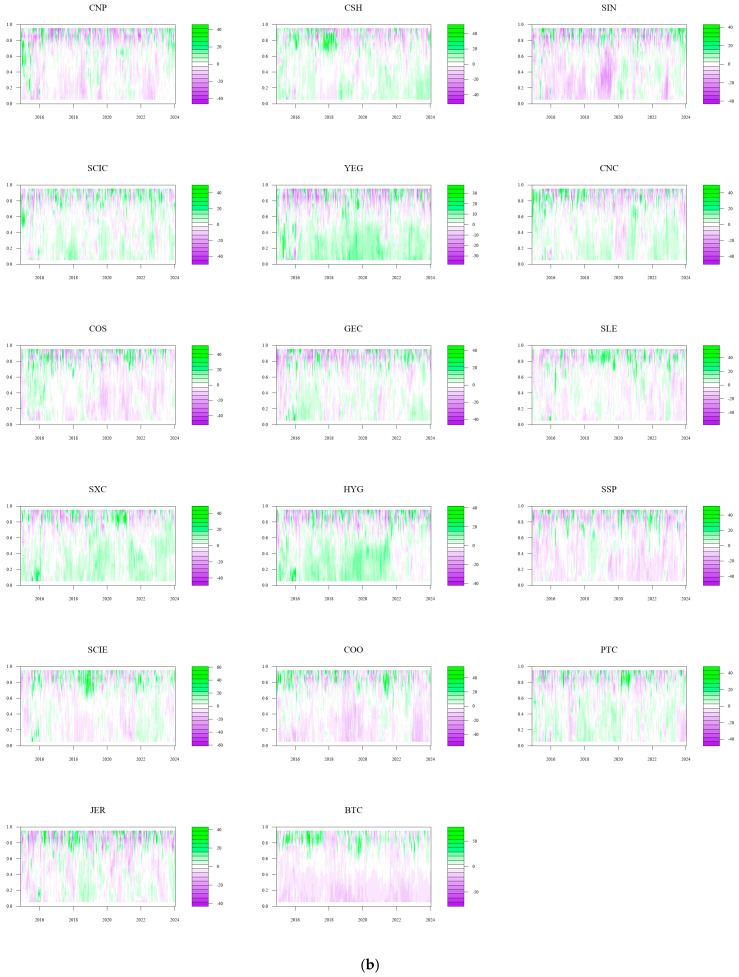

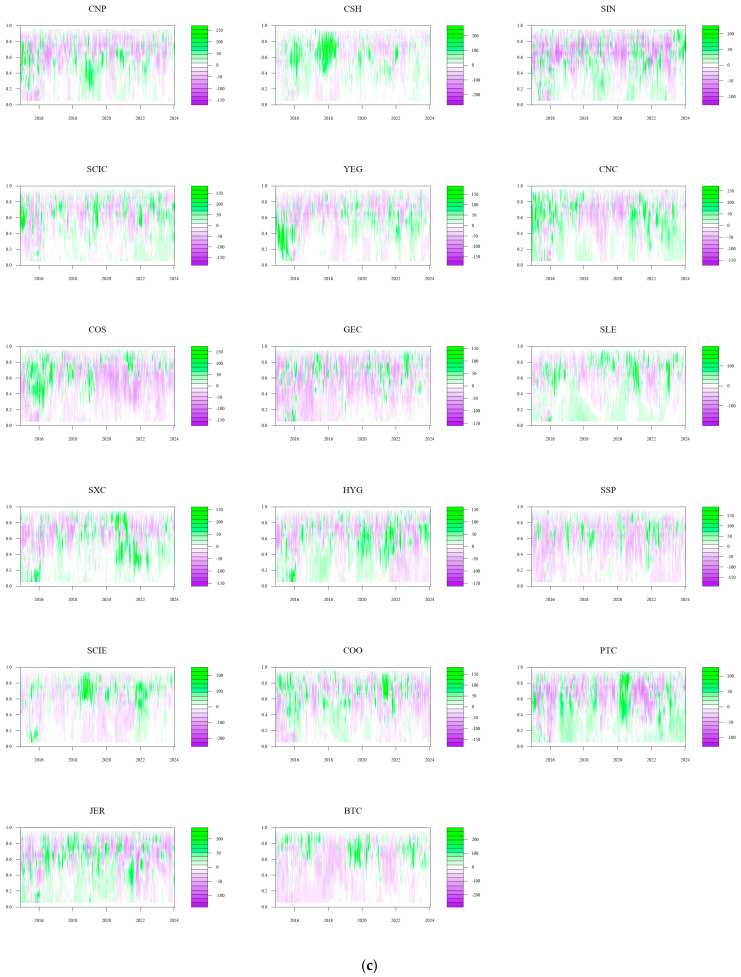
(**a**) Short−term tail risk net spillover effect between the cryptocurrency and energy markets. (**b**) Medium−term tail risk net spillover effect between the cryptocurrency and energy markets. (**c**) Long−term tail risk net spillover effect between the cryptocurrency and energy markets.

## Data Availability

The original contributions presented in this study are included in the article. Further inquiries can be directed to the corresponding author.

## Appendix A. Descriptive Statistics

**Table A1:** Descriptive statistics of logarithmic returns.

Variable	Mean	Minimum	Maximum	Variance	Skewness	Kurtosis	Jarque-Bera
CNP	−0.000027	−0.105244	0.095636	0.000300	0.046049	7.296687	5415.206
CSH	0.000353	−0.142997	0.095487	0.000474	−0.371965	6.188202	3950.9369
SIN	0.000060	−0.105807	0.095630	0.000277	−0.353348	6.942405	4952.237
SCIC	0.000660	−0.124568	0.095993	0.000742	−0.224247	2.873445	860.9597
YEG	0.000432	−0.591336	0.095864	0.001120	−2.327731	41.168308	174,459.6079
CNC	0.000371	−0.107631	0.095891	0.000649	−0.201124	4.274696	1875.5766
COS	−0.000157	−0.105631	0.095737	0.000663	−0.108174	3.375334	1164.249
GEC	0.000017	−0.113759	0.096159	0.000641	−0.142312	3.749895	1439.1314
SLE	0.000362	−0.245087	0.096026	0.000873	−0.349495	4.012301	1687.6606
SXC	0.000215	−0.291028	0.096100	0.000887	−0.431423	6.021678	3763.6358
HYG	0.000178	−0.476967	0.096100	0.000898	−1.71198	28.053193	81,182.1093
SSP	−0.000036	−0.105956	0.096522	0.000556	−0.051379	5.868337	3503.7159
SCIE	0.000565	−0.181092	0.096627	0.001117	−0.155580	2.106496	461.8347
COO	−0.000146	−0.105770	0.096074	0.000550	−0.294255	4.622766	2209.225
PTC	0.000384	−0.124313	0.096163	0.000827	−0.200230	2.875170	857.8261
JER	0.000042	−0.291108	0.095952	0.000780	−0.639923	8.073070	6793.8023
BTC	0.001550	−0.848829	1.474180	0.003496	4.700465	180.342682	3,314,272.4956

## Appendix B. Time–Frequency Domain Tail Risk Net Spillover Effect Between Cryptocurrency and Energy Markets

Figure A1 shows the dynamic variations in the net spillover of tail risk of Bitcoin and energy companies based on various quantiles. Among these, purple indicates a negative net spillover impact from tail risk, green indicates a positive net spillover effect from tail risk, and purple to green indicates a transition from a negative to a positive net spillover effect.

The time–domain analysis is extended to the frequency domain in Figure A2a–c, where it is broken down into the dynamic variations of net spillover effects under various quantiles across varying time horizons.
